# How do pigs deal with dietary phosphorus deficiency?

**DOI:** 10.1017/S0007114520000975

**Published:** 2020-03-16

**Authors:** Maciej M. Misiura, João A. N. Filipe, Carrie L. Walk, Ilias Kyriazakis

**Affiliations:** 1 Agriculture, School of Natural and Environmental Sciences, Newcastle University, Newcastle upon Tyne NE1 7RU, UK; 2 Rowett Institute of Nutrition and Health, University of Aberdeen, Aberdeen AB25 2ZD, UK; 3 AB Vista, Marlborough SN8 4AB, UK; 4 Institute for Global Food Security, Biological Sciences Building, Queen’s University, Belfast BT9 5DL, UK

**Keywords:** Phosphorus, Retention, Models, Growth, Pigs

## Abstract

Feeding strategies for growing monogastric livestock (particularly pigs) must focus on maximising animal performance, while attempting to reduce environmental P load. Achieving these goals requires a comprehensive understanding of how different P feeding strategies affect animal responses and an ability to predict P retention. Although along with Ca, P is the most researched macromineral in pig nutrition, knowledge gaps still exist in relation to: (1) the effects of P feed content on feed intake (FI); (2) the impact of P intake on body composition; (3) the distribution of absorbed P to pools within the body. Here, we address these knowledge gaps by gathering empirical evidence on the effects of P-deficient feeds and by developing a predictive, mechanistic model of P utilisation and retention incorporating this evidence. Based on our statistical analyses of published literature data, we found: (1) no change in FI response in pigs given lower P feed contents; (2) the body ash–protein relationship to be dependent upon feed composition, with the isometric relationship only holding for pigs given balanced feeds and (3) the priority to be given towards P retention in soft tissue over P retention in bones. Subsequent results of the mechanistic model of P retention indicated that a potential reduction in P feeding recommendations could be possible without compromising average daily gain; however, such a reduction would impact P deposition in bones. Our study enhances our current knowledge of P utilisation and by extension excretion and could contribute towards developing more accurate P feeding guidelines.

Due to the economic and environmental importance of P^(1–3)^, the present and future feeding strategies for growing monogastric livestock must focus on minimising P excretion as well as on maximising animal performance. This is particularly the case for growing and finishing pigs, for which various methods for reducing the environmental load of P have been proposed, including the use of exogenous phytase enzymes to improve P digestion^(4–9)^. In addition, there are recommendations to lower P levels in diets^(10)^ to address sustainability concerns associated with P overfeeding. For example, the National Research Council (NRC)^(11)^ suggests that pigs are fed 15 % below the estimated P requirements, provided that they are not destined for breeding stock. Nevertheless, many commercial pig feeding formulations still contain excess P levels as a safety buffer to avoid the potential production losses and welfare concerns resulting from P underfeeding^(12)^, such as reduced average daily gain (ADG)^(13)^ and inadequate bone mineralisation and development^(14–20)^. The widespread use of such safety margins reflects uncertainty in the quantification of short-term responses to deficient dietary P. Specifically, when animals are given feeds with deficient P content:

Q1. Do they modify their feed intake (FI)?
Q2. How is the relationship between bone mineralisation and muscle tissue affected?
Q3. Are the P intake resources allocated differently within the body?

Currently, there are partial and sometimes conflicting literature reports on pigs and other monogastric livestock on the answers to these questions, which have potentially profound impacts on diet formulation and the subsequent nutrient excretion. Here, we address these questions with a focus on commercial pigs.

Further to this general relevance, the above questions are also pertinent for existing mechanistic models of P utilisation, which are instrumental in efforts to increase P feed efficiency in commercial pig production systems. Currently, such models make specific assumptions about the processes associated with Q1–Q3 that may or may not be valid, or whose validity may hold only for a specific range of conditions. In relation to the FI response to P-deficient feeds (Q1), only Symeou *et al.*^(21)^ and Létourneau-Montminy *et al.*^(22)^ can predict FI using a relationship to modelled traits. Only Symeou *et al.*^(21)^ have allowed for adaptable FI depending on the nutrient composition. Other relevant models^(23–25)^ use FI as a direct input, which can restrict their applicability in scenarios where such FI data are unavailable.

Most of the current growth models^(11,21,26–30)^ in pigs assume that the body pools of ash (located mainly in bones) and muscle tissue grow at a constant proportion to each other irrespective of nutrient composition (Q2). While under non-limiting feeding conditions, this relationship is well documented^(31)^, it is unclear what happens when pigs are given feeds that are deficient in either P or protein. In relation to the allocation of deficient P intake (Q3), the predictive models of P utilisation differ on whether this allocation changes under different nutritional scenarios. Symeou *et al.*^(21)^ assume constant proportionality between P and protein retention even in cases of mineral deficiency, while Létourneau-Montminy *et al.*^(22)^ allow for a prioritisation towards P in soft tissue under deficient mineral intake. The latter model requires depicting P kinetics in the body of an animal as a multi-pool process.

Given differences in these assumptions and a lack of conclusive literature evidence, the objectives of this study were to: (1) address Q1–Q3 through a meta-regression analysis of the current literature evidence; (2) incorporate new data-based answers to Q1–Q3 in a revised model of P requirements and retention in pigs^(21)^ and (3) predict pig responses to diets of different levels of P.

The outcomes of this paper were expected to enhance our understanding of P nutrition on animal growth and body composition and could contribute towards issuing more accurate P feeding guidelines in the future as well as increasing our ability to quantify the economic and environmental consequences associated with alternative P feeding strategies.

## Materials and methods

### Data collection and data processing

To address the three main research questions (Q1–Q3), we analysed the relevant published literature data, which was collected according to the pre-defined inclusion criteria. The applied inclusion criteria differed for each research question and are described in more detail below. Note that there was no requirement for ethical approval, since the data were obtained from peer-reviewed articles, in which ethical approval was already obtained by the trial investigators.

#### Data on feed intake

To ascertain if changes in feed P levels alter the voluntary daily FI (kg/d) response, we focused on identifying experiments designed to study the P requirements of growing (weaning to 50 kg body weight (BW)) and finishing pigs (50–100 kg BW), which satisfied inclusion criteria outlined in Table 1. A total of fifteen studies were identified (see online Supplementary material for more details); the most frequent reasons for rejection were: (1) feeding was not *ad libitum*; (2) dietary treatments included exogenous phytase (exogenous phytase alters the amount of digestible P and hence can alter the response to the perceived P deficiencies); (3) insufficient number of dietary treatments was considered (as the estimated P requirements for maintenance and growth are imprecise, especially across different pig breeds, several P levels are needed to find the point of response, if any) and (4) the required data were missing (e.g. BW measurements per dietary treatment).

**Table 1.** Inclusion and exclusion criteria used to select studies for the statistical analysis of feed intake regulation

Component	Inclusion	Exclusion
Study design	Quantitative, *in vivo*: dose–response study	Descriptive; qualitative; *in silico*; *in vitro*
Population	Growing (i.e. pigs that overcame stress associated with weaning) and finishing (50–100 kg initial live weight) pigs irrespective of breed	New-born piglets; weaning pigs; sows
Subpopulation	Barrows (castrated males); boars (entire males); gilts	–
Treatment	A minimum of four homogenous feeds differing in their P concentration (as the estimated P requirements for maintenance and growth are imprecise, especially across different pig breeds, several P levels are needed to find the point of response, if any) Once assigned to a given feed, pigs were fed *ad libitum* throughout the trial duration	Other nutrients (energy, protein, minerals other than P) varied and supplied below the estimated requirement Exogenous phytase supplementation Pigs given choice to select their own diet Pigs fed an allowance
Primary outcome	ADFI for each feed treatment group	–
Additional reported data	Average initial and final body weights for each treatment group Sufficient feed composition information to estimate STTD P	–

ADFI, average daily feed intake; STTD P, standardised total tract digestible P.

The following data were extracted: (1) study information (first author, publication year and location); (2) feed composition characteristics (metabolisable energy, crude protein, P and Ca contents); (3) mean values with their standard errors of the initial and final BW, average daily FI (ADFI) and either ADG or experimental duration.

#### Data on ash, phosphorus and protein weights in the body

To quantify the relationship between the relevant body components, published data, comprising the serial measurements of body P and/or ash, and protein weights in growing and finishing barrows, boars and gilts were eligible for inclusion if: (1) feeds given to pigs could be categorised as: (i) nutritionally balanced with respect to all nutrients, meeting or exceeding the appropriate nutritional guidelines available at the time of each publication^(11,32–34)^ (100 % or above of the relevant requirement); (ii) P-deficient (below the relevant requirement, with a minimum threshold set at 50 %), but balanced with respect to energy and protein as defined by the trial investigators and (iii) protein-deficient (below the relevant requirement, with a minimum threshold set at 50 %), but balanced with respect to energy and essential minerals as defined by the trial investigators; (2) once assigned to a dietary treatment, pigs were given the same feed throughout the experimental period, until the slaughter weight was reached and (3) pigs were not exposed to any nutritional deficiencies prior to the start of each trial.

The following data were extracted: (1) study information; (2) mean values with their standard errors of the reported P and/or ash, and protein content of the empty BW (eBW). The P–protein BW database consisted of data originating from eleven studies, whereas the ash–protein BW database included data from twenty-five articles (see online Supplementary material).

#### Data on phosphorus partitioning in body pools

In pigs kept under non-limiting conditions, approximately 77–80 % of body P are found in bones^(35,36)^, with the remainder of body P located in soft tissue^(37)^. Based on the initial scoping of the literature^(38,39)^, it was determined that the *a priori* aim of collecting and analysing published literature data on the separate P contents of soft tissue and bones of pigs given P-adequate or P-deficient feeds was not feasible due to insufficient data. An alternative approach was set out, and analysis was performed on the subset of the serial slaughter database described above, that is, with simultaneous measurements of P, Ca and eBW for each dietary treatment. This information was used to derive plausible estimates of P_bone_ weights by utilising the following well-established relationships: (1) 99 % of Ca is found in bones^(36)^ and (2) bone Ca is deposited together with P as hydroxyapatite at a constant Ca:P ratio of 2·16:1^(40)^. Hence, P_bone_ was calculated according to the following formula:
(1)
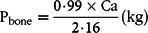


This quantity was estimated in all eleven included studies (*n* 136/136 data points).

### Data analyses

#### Feed intake

The reported ADFI was chosen as the dependent variable in assessing FI regulation. However, since ADFI varies with body size, it was necessary to avoid biasing conclusions regarding the effects of diets on FI regulation^(41)^. Consequently, the BW-specific ADFI, (scaled ADFI per kg of current BW) was chosen as a way of comparing the FI responses, given the approximately linear relationship between FI and BW over the weight range examined^(42)^. Since our data set consisted of BW measurements at the start and end of each trial, with no other intermediate BW measurements, the BW-specific ADFI was evaluated by dividing each reported ADFI by the midpoint of the BW range for each dietary treatment group in each study. Alternative analyses were also performed with unscaled ADFI and ADFI scaled either per kg of BW^0·75^ or BW^0·66^ to test if the analysis was affected by other common scaling choices^(43)^.

The independent variable of interest was P feed content of each selected dietary treatment group, expressed on a digestible basis as standardised total tract digestible (STTD)^(11,44)^ values. If STTD P feed content was not reported, this quantity was estimated for each dietary treatments on the basis of the reported list of ingredients used to formulate the aforementioned treatments and the nutritional data from the NRC feed composition tables^(11)^. The STTD *P* values needed to be estimated for eleven out of fifteen studies (*n* 66/97 data points).

Meta-regression^(45–47)^ was utilised to examine the impact of different P feed contents on FI. Procedures outlined in Misiura *et al.*^(48)^ were followed. First, the existence of random effects arising from data heterogeneity was formally assessed using the likelihood ratio test between an intercept-only model and an alternative nested model with an additional random term associated with each selected study^(46)^. The result of a *χ*^2^ test with one df provided strong evidence against the null model and hence supported the addition of this random effect. Consequently, a linear mixed effects regression (LMER) model was fitted to the data. In this model, the scaled ADFI was the dependent variable; the independent variables were the STTD P feed content represented as a continuous fixed effect and each study represented as a random effect. Each fitted observation was weighted by the inverse of its associated-group-study variance (variance, calculated from the reported SEM and sample sizes) to account for any potential heteroscedasticity.

Model fitting was performed with the nlme (version 3.1–3.137)^(49)^ and metafor (version 2.0-0)^(50)^ packages in R (version 3.5.3)^(51)^, where the restricted maximum likelihood method is used to derive variance and covariance components. Conditional *F* tests^(52)^ on the restricted maximum likelihood method variance estimates were implemented to test the effect of STTD P feed content on the scaled ADFI at a 0·05 significance level. Validity of the LMER model was tested by assessing QQ plots of the standardised residuals and scatter plots of the standardised residuals against the fitted values generated separately for the fixed and the random parts of the statistical model. These diagnostic plots did not reveal any major deviations from normality or heteroscedasticity of the fixed and random effects residuals and hence did not invalidate the LMER model assumptions^(52)^.

#### Relationship between ash–protein and phosphorus–protein body weights

To assess if there were modifications in the relative body composition across nutritional scenarios, we hypothesised that during growth, the ash and P BW (kg) were related to the protein weight (kg) via allometry^(53,54)^:
(2)
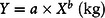

where *a, b* > 0 captured the effect of the nutrition scenarios and *Y* = {*P*, ash}. Based on the value of the allometric exponent *b*, the following three main cases were considered: (1) *b* = 1: proportional relationship (isometry), where the weight of P or ash was directly proportional to protein; (2) *b* > 1: the weight of P or ash increased at a faster relative rate than protein weight (positive allometry); (3) *b* < 1: the weight of P or ash increased at a slower relative rate than protein weight (negative allometry). The above power law equation (2) was transformed using the natural logarithm (ln):
(3)
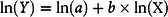

and fitted to the data using linear regression in R (version 3.5.3)^(51)^ for each of the following nutritional scenarios: (1) P–protein relationship for balanced feeds and for P-deficient feeds and (2) ash–protein relationship for balanced feeds, for P-deficient feeds and for protein-deficient feeds. Owing to insufficient data, it was not possible to investigate the P–protein relationship for protein-deficient feeds. For each study, feeds were classified as balanced, P- or protein-deficient on the basis of the authors’ description, which was reviewed against the appropriate nutritional guidelines cited by the authors, prevailing at the time of the experiment (section Feed intake).

The associated-group-study variances were used as weights to account for potential heteroscedasticity and measurement error associated with each reported observation. Here, the addition of random study effects was not justified based on insignificant *P* values of *χ*^2^ tests of the intercept-only models and alternative nested models with study represented as a random effect.

Estimates of the scaling exponent *b*, together with the associated 95 % CI, were used to detect any differences across the nutritional scenarios in *t* tests of the null hypothesis that *b* = 1. Model validity was assessed using QQ plots of the standardised residuals and scatter plots of the standardised residuals against the fitted values. Goodness of fit of statistical models was quantified through the coefficient of determination (*R*^2^)^(55,56)^.

Since P, ash and protein weights had observation error and could be used interchangeably as either *y*-axis or *x*-axis variables, model fitting was also performed by reversing the two variables of interest^(57)^. As a further assurance that the parameter estimates were not influenced by the assumption of linear regression that the *x*-axis variable is error-free, model fitting was also performed via reduced major axis regression^(58,59)^ using the smatr package (version 3.4-8)^(60)^, which accounted for the error in both *y*-axis and *x*-axis variables.

#### Phosphorus partitioning in body pools

To address our third question, body P was expressed as a sum of the amounts located in soft tissue and bones^(40)^:
(4)
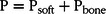


While it is established that in pigs given balanced feeds, P_bone_ weight should account for approximately 77–80 % of the total P weight^(35,36)^, with the remaining P located in soft tissue^(37)^, it is unclear if this relationship also holds true for P-deficient feeds.

Regression was used to quantify the relationship between body P and P_bone_ and determine if P_bone_ is a constant or variable proportion of P in each of the feeding schedules (either balanced feeds or P-deficient feeds). The dependent variable was the percentage of P in bone (P_bone_/P × 100, P_bone%_). The independent variable was P weight per unit of eBW (P; g/kg of eBW). Beta regression^(61)^ was chosen to analyse the data using the *betareg* package (version 3.1-1)^(62)^ in R (version 3.53)^(51)^ with each observation weighted by the inverse of its associated-group-study variance. The beta regression models were parameterised in terms of a beta probability density with mean *μ* and precision φ accounting for any potential dispersion within the data according to Cribari-Neto & Zeilis^(61)^. The independent variable was tested for significance in both mean and precision components of the beta regression model. Model validity was diagnosed using scatter plots of the residuals against the fitted values and a half-normal plot of residuals^(61)^. Goodness-of-fit model was evaluated using a pseudo-*R*^2^ (squared correlation of linear predictor and link-transformed response^(62)^).

### Model of body mass growth and phosphorus retention

**Fig. 1.**
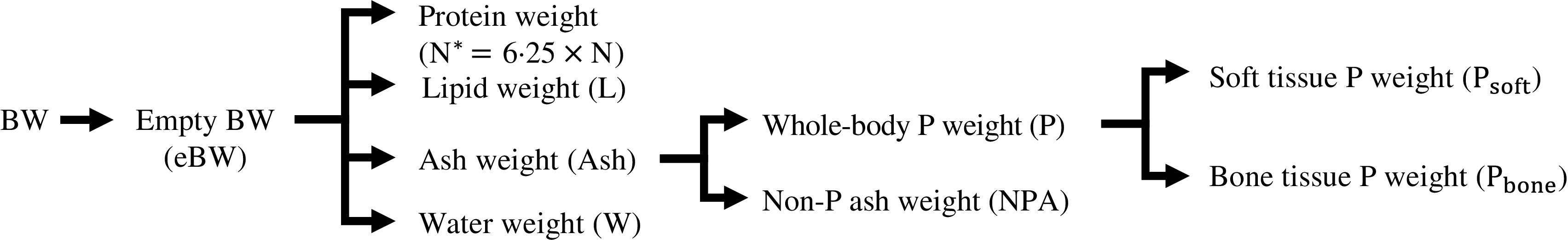
Basic animal description, demonstrating the partitioning of live body weight (BW) into the main body components of growing and finishing pigs.

A dynamic, mechanistic model simulating body mass growth and P retention of an individual pig was developed to simulate the effects of different STTD P^(11,44)^ feed contents (g/kg) on animal performance. The model stems from the approach by Symeou *et al.*^(21)^ and Wellock *et al.*^(27)^, where daily growth was estimated from the predicted FI, but constitutes a new development. Once the gut fill was accounted for, eBW (kg) of a pig was expressed as a sum of the four main body components: protein (N* = 6·25× N) (kg), lipid (L) (kg), water (W) (kg) and ash (kg) (Fig. 1). Pig phenotypes were characterised in terms of the daily growth rate (B), mature protein (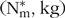 and mature lipid (L_m_, kg) weights. A detailed list of equations used to describe the relationships between variables is given in the online Supplementary material.

#### Balanced feeds

A healthy pig, given balanced feed and kept in a thermally neutral environment, was expected to achieve the maximum growth determined by its genotype^(63)^. The maximum growth of protein and L weights was represented by Gompertz^(64)^ functions of age, parameterised by mature weight (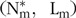) (kg) and a rate (B)^(65)^:
(5)
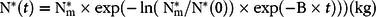

(6)
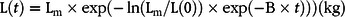

where *t* is the time in d from a given age, N*(0) and L(0) were estimates of the initial protein and L weights. The maximum daily retention of protein (kg/d) at time *t* is the derivative of equation (5):
(7)
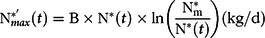


The actual retention of protein and L (kg/d), determined by the FI and the maintenance requirements, and the retention of ash, W and P in terms of the actual protein retention were implemented as in Wellock *et al.*^(27)^.

#### Phosphorus-unbalanced feeds

The actual growth (which could be different from maximum growth) was determined by the predicted feed consumption. In the context of pigs given *ad libitum* access to feeds that differ in P content but were balanced with respect to all other nutrients, three candidate FI responses: (i) decrease; (ii) no change and (iii) increase, were proposed and tested.

Theory 1: pigs reduce their FI according to the magnitude of P deficiency and hence P feed content is the main determinant of the actual, observed FI. Theory 2: FI is controlled only by the energy needed to support the potential growth and hence FI is unaffected by P feed content:
(8)
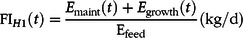

where E_maint_(*t*) (MJ/d) is the daily energy requirement for maintenance at time *t*, E_growth_(*t*) (MJ/d) is the energy associated with the maximum protein and L daily retentions, and E_feed_ is the energy content of the feed (MJ/kg); energy contents were expressed in terms of effective energy^(66)^. Theory 3: pigs given P-deficient feeds attempt to increase their daily FI relative to pigs supplied with adequate P feed levels, implying that FI is controlled by the P requirements for maintenance and growth. Examining the three theoretical FI responses was equivalent to testing whether the slope of the fitted LMER model (section Model of body mass growth and phosphorus retention) was statistically different from zero. If the slope was statistically significant from zero, then direction of the slope was used to determine if FI was either increasing or decreasing with changes in P feed content. Hence, the actual FI (*t*) function (kg/d) incorporated in the mechanistic model was the one supported by the statistical analyses of the FI data (section Data analyses). The actual protein retention, N*’(t), was determined by the actual FI function used. The actual daily L retention was calculated as follows: if remaining energy was available from intake:
(9)
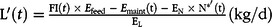

otherwise L’(*t*) = 0; where E_N_ and E_L_ are the energy used (and expressed in effective energy scale) per kg of protein and L retained, respectively. The absorbed P intake at time *t*, PI(*t*) (kg/d), obtained from the predicted FI(*t*) was partitioned towards maintenance 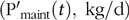
and body growth (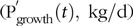):
(10)
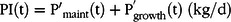

with 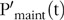 assumed to be proportional to the current body protein, N*(t) ^(67)^:
(11)
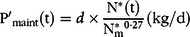

where *d* is an appropriate scaling coefficient^(21)^. Maintenance requirements for P were expressed as a function of protein to account for: (1) differences in body composition, with a focus on variability in body fat (lipid tissue contains only a negligible P content^(35,68)^) and (2) a close relationship between P maintenance and protein turnover^(36)^.

The actual 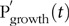
was the difference between PI(*t*) and 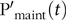, assuming efficiencies of 0·94^(21)^ (eff_P_) for 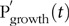 and 1·00^(21)^ for 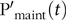

(12)
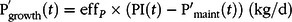

provided this did not exceed 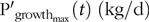
defined as (21):
(13)
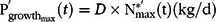

otherwise:
(14)
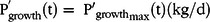

where D is evaluated using the results of the statistical analysis of the P–protein relationship (section Model of body mass growth and phosphorus retention).

To account for the two pools of P within the body, 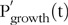 was expressed according to equation (4) as:
(15)
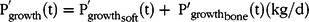


Partitioning of PI(*t*) towards these two pools was hypothesised to occur either: (1) as a fixed proportion of the ‘P profile in the body’, that is, amounts of P located in soft tissue and bones reported for growing and finishing pigs fed balanced feeds^(35,36)^ under a constant ratio of allocation to the two pools; or (2) according to a prioritisation of resources towards 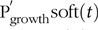
to support the maximum protein growth^(37)^, with any remaining P being allocated to 
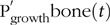
. The partitioning hypothesis used in the mechanistic model of P retention was the one supported by the statistical analysis of the P partitioning (section Model of body mass growth and phosphorus retention).

In cases where 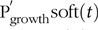 was below the level needed to support 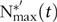, the actual 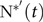 was decreased and calculated by incorporating the results of the statistical analysis of the P–protein relationship (section Model of body mass growth and phosphorus retention). The W–protein relationship was assumed to be unchanged from section Balanced feeds, but the ash–protein relationship was informed by the previous statistical analysis (section Data analyses).

### Model exploration

The mechanistic model, incorporating the data-supported mechanism of FI regulation, the P–protein and ash–protein relationships in the body, and the partitioning of P in body pools, was used to generate predictions over a range of pig phenotypes and feeds. Each simulation yielded predicted performance of an individual pig from one of the considered phenotypes (section Phenotype) given *ad libitum* access to a single feed with a STTD P content set to one of the investigated levels (section Feed). All simulations were carried out from an initial BW of 25·0 kg for a period of 42 d. The following model outputs were produced for each simulated scenario: (1) ADFI (kg/d); (2) ADG (kg/d); (3) average feed conversion ratio over the duration of the experimental period and (4) final BW (kg). The effects of P-deficient feeds on animal body composition were evaluated via the predicted daily protein retention, daily P retention and P_soft_ and P_bone_ retention.

#### Phenotype

To illustrate the effects of pig phenotypic differences on P retention and overall animal performance, three pig lines were considered using estimated pig phenotype parameters for the UK pigs based on Large White × Landrace crosses, characterised by the British Society of Animal Science at the time of publication, as being^(34)^: (1) fast-growth pig, 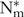 = 50·0 kg, L_m_ = 55·0 kg, B = 0·0125; (2) intermediate-growth pig, 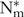
= 40·0 kg, L_m_ = 48·0 kg, B = 0·0118; (3) commercial pig, 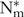 = 30·0 kg, L_m_ = 39·0 kg, B = 0·0110.

#### Feed

The baseline kg of feed contained 13·6 MJ of metabolisable energy, 174 g of crude protein, 11·1 g of lysine and 3·10 g of STTD P, which was considered to provide adequate quantities of these nutrients to support maximum lean tissue growth in pigs based on Large White × Landrace crosses^(11,69)^; the baseline feed was assumed to be abundant in vitamin D, Ca and other essential minerals and did not contain any exogenous phytase enzymes. To illustrate the effects of different P feed levels on daily P retention, fourteen feeds were considered with STTD P feed content ranging from 1·60 to 5·50 g/kg of feed, which corresponded to 50–180 % of the NRC guidelines^(11)^ for 25–50 kg pigs. All other feed components remained unchanged.

### Model validation

The performance of the proposed mechanistic model of pig growth and P retention was compared with the published literature data excluded at random from the statistical analyses described in section Data analyses. To be eligible for validation purposes, published studies had to simultaneously satisfy the inclusion criteria outlined in section Data on feed intake and Data on ash, phosphorus and protein weights in the body. *A priori*, it was decided that the majority of the relevant data (approximately 80 %) should be utilised for the purposes of the statistical inference, with the remainder used for model validation^(70)^. To minimise bias, sampling of the data sets was carried out using algorithm for random sampling without replacement in R (version 3.5.3)^(51)^.

#### Estimation of pig phenotype parameters

Since the pig phenotype parameters (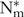, L_m_ and B) required to run the mechanistic model were not reported in the *in vivo* experiments selected for the model validation, these parameters had to be estimated from the reported data. Parameter estimation was carried out by utilising the concepts of inverse modelling^(71,72)^ and the methodology described in Wellock *et al.*^(73)^. For each selected study, Gompertz curves for N*(t) and L(t) were fitted to the following data which corresponded to a dietary treatment, which maximised pig performance in terms of ADG: (1) initial BW; (2) final BW; (3) cumulative energy intake, calculated from the reported ADFI, length of an experimental period and metabolisable energy feed content. Data fitting was performed in R (version 3.5.3)^(51)^ with the minpack.lm package (version 1.2-1)^(74)^ using the Levenberg–Marquardt algorithm.

#### Validation procedure

Model validation was performed by assessing whether the mechanistic model was able to recreate the empirical results reported in each study. The reported feed composition (STTD P values needed to be estimated for two out of three studies (*n* 10/16 data points)) and estimated pig phenotype parameters were used as inputs in the model. The following outputs were generated to evaluate performance of the model: (1) daily P retention (g/d); (2) daily protein retention (g/d) and (3) ADFI (kg/d). These variables were chosen, as they corresponded to the reported quantities in the selected studies. A three-step validation procedure was utilised. First, graphical comparison of the model outputs and observed values was used to assess their qualitative agreement^(73)^. Second, the scaled residuals (r_i_), expressed as the difference between the reported (y_i_) and predicted (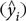) data scaled by the observed standard deviation, were calculated for each of the four variables of interest:
(16)
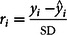

where *i* corresponds to each dietary treatment. Plots of these scaled residuals against model predictions were generated to identify any systematic trends and identify any potential biases in the model. Third, the following scale-independent quantitative measures of the model accuracy were used for each predicted variable: (1) the mean absolute percentage error (MAPE)^(75)^:
(17)
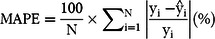

where *N* is the total sample size; (2) *R*^2^, defined for each variable in the usual way, as:
(18)
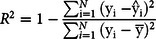

where 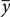 is the sample mean. For MAPE, percentage values closer to zero indicate smaller predictive error. For *R*^2^, values closer to one indicate that the model explains most of the variation in the data.

## Results

### Feed intake

**Fig. 2.**
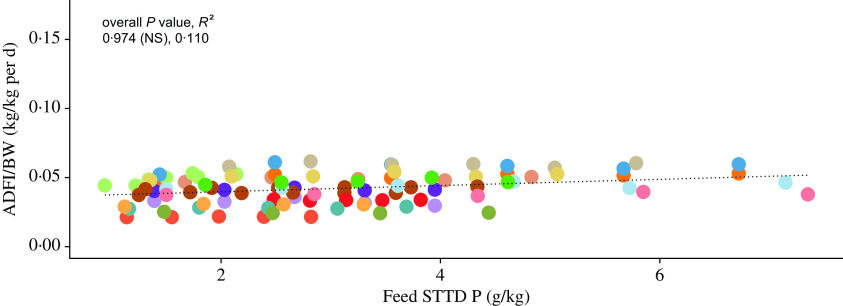
Body weight (BW)-scaled average daily feed intake (ADFI/BW; kg/kg per d) against feed standardised total tract digestible (STTD) phosphorus content (g/kg): individual data points (*n* 97) represent mean values for each considered dietary treatment reported in fifteen studies (see online Supplementary material for a list of included studies). The dotted line represents the overall predicted effect of feed STTD phosphorus content on the scaled ADFI. Probability (*P*) and coefficient of determination (*R*^2^) values are given for the overall fit and for fit within the included experiments. * *P* < 0·05; ** *P* < 0·01; *** *P* < 0·001. Data: first author, year (*P*, *R*^2^): 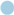, Alebrante, 2011 (0·179 (NS), 0·248); 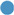, Arouca, 2012a (0·464 (NS), −0·074); 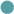, Arouca, 2012b (0·319 (NS), 0·095); 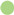, Baker, 2013 (0·007 (**), 0·751); 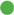, Bunzen, 2012 (0·261 (NS), 0·185); 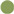, Campos, 2012 (0·444 (NS), −0·035); 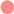, Carter, 1998 (0·542 (NS), 0·153); 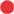, Hastad, 2004a (0·894 (NS), −0·324); 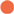, Hastad, 2004b (0·241 (NS), 0·220); 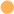, Mavromichalis, 1999 (0·362 (NS), 0·110); 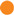, Saraiva, 2009 (0·130 (NS), 0·343); 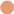, Saraiva, 2011 (0·361 (NS), 0·038); 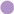, Saraiva, 2012a (0·340 (NS), 0·065); 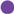, Saraiva, 2012b (0·678 (NS), −0·246); 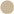, Zhai, 2013a (0·971 (NS), −0·250); 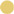, Zhai, 2013b (0·175 (NS), 0·254); 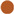, Zhai, 2013c (0·183 (NS), 0·087).

The scaled ADFI (kg/kg of BW per d) against the STTD P feed content (g/kg of feed) from the included literature data is shown in Fig. 2 (see online Supplementary material for a list of included studies). On the basis of the fitted LMER model, the STTD P feed content had a non-significant effect on the scaled ADFI (95 % CI −0·0138, 0·0190; *P* > 0·05). This result suggests that pigs did not modify their FI response when given P-deficient feeds, thus giving support towards Theory 2 outlined in section Feed. Similarly, the STTD P feed content had a non-significant effect on the unscaled ADFI (kg/d) and of the alternative versions of the scaled ADFI (either kg/kg of BW^0·75^ per d or kg/kg of BW^0·66^ per d) (online Supplementary material).

### Relationship between ash–protein and phosphorus–protein body weights

#### Phosphorus–protein relationship

**Fig. 3.**
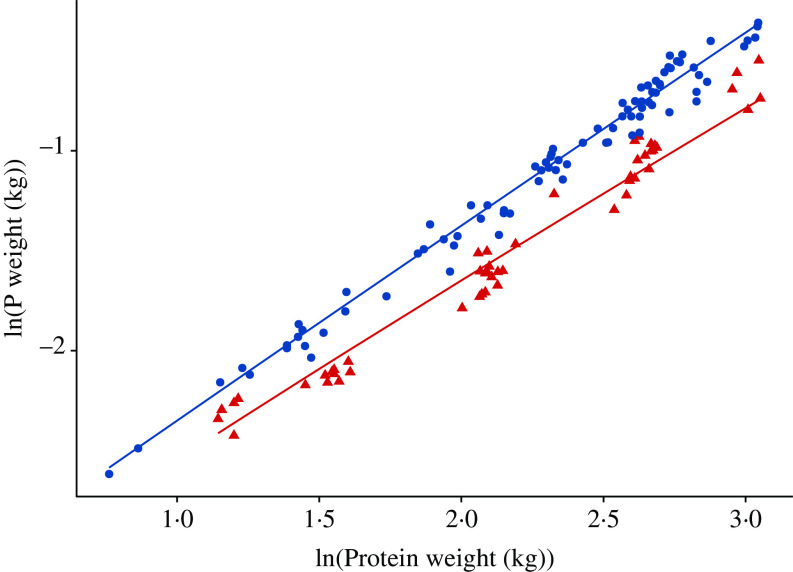
Relationship between body phosphorus weight and body protein at the time of slaughter weights in growing and finishing pigs; individual data points (*n* 136) represent mean values for each considered dietary treatment reported in eleven studies (see online Supplementary material for a list of included studies). The data were expressed on the natural logarithmic (ln) scale, under the following two *ad libitum* feeding schedules: (1) pigs were given nutritionally balanced feeds; (2) pigs were given phosphorus-deficient feeds. Data: 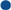, balanced feed; 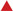, phosphorus-deficient feed.

The relationship between body P (kg) and protein (kg) weights at the time of slaughter from the included literature data (see online Supplementary material for a list of included studies) is visualised in Fig. 3. Results of the weighted least squares regression of P weight (kg) on protein weight (kg) for pigs given either nutritionally balanced or P-deficient feeds are summarised in Table 2. For pigs given balanced feeds, the allometric exponent b was 0·982 (95 % CI 0·954, 1·01); hence, the P–protein isometry hypothesis (*b* = 1) could not be rejected (*P* > 0·05). For pigs supplied with P-deficient feeds, the estimated allometric exponent *b* was 0·924 (95 % CI 0·878, 0·960), thus rejecting the isometry hypothesis (*P* < 0·01) in favour of allometric scaling (*b* < 1). High *R*^2^ values (0·982 for balanced feeds and 0·972 for P-deficient feeds) indicated a good model fit to the data. Repeating model fitting using the two variables in reversed roles (e.g. with P as the *x*-axis variable) led to analogous conclusions. Performing model fitting via reduced major axis regression of P weight on protein weight yielded similar findings (online Supplementary material).

**Table 2.** Regression estimates, standard errors, 95 % confidence intervals and coefficients of determination (*R*^2^) for the fitted relationship: 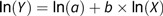, where *Y* was the body phosphorus weight and *X* was the body protein weight under the following two *ad libitum* feeding schedules: (1) pigs were given nutritionally balanced feeds; (2) pigs were given phosphorus-deficient feeds* (Regression estimates, standard errors, 95 % confidence intervals and coefficients of determination)

Parameter	Feed class	Estimate	se	95 % CI	*P*	*R*^2^
ln(*a*)	Balanced	−3·35	0·0319	−3·41, −3·29		0·972 (P-deficient)
	P-deficient	−3·51	0·0490	−3·60, −3·41		
*b*	Balanced	0·982	0·0138	0·954, 1·01	0·193	0·982 (balanced)
	P-deficient	0·924	0·0226	0·878, 0·960	0·00146	

* Probability values were given for the direct test of whether the estimated relationship was isometric (*b* = 1). A total of 136 individual data points representing mean values for each considered dietary treatment reported in eleven studies (see online Supplementary material for a list of included studies) were used in the analysis.

#### Ash–protein relationship

**Fig. 4.**
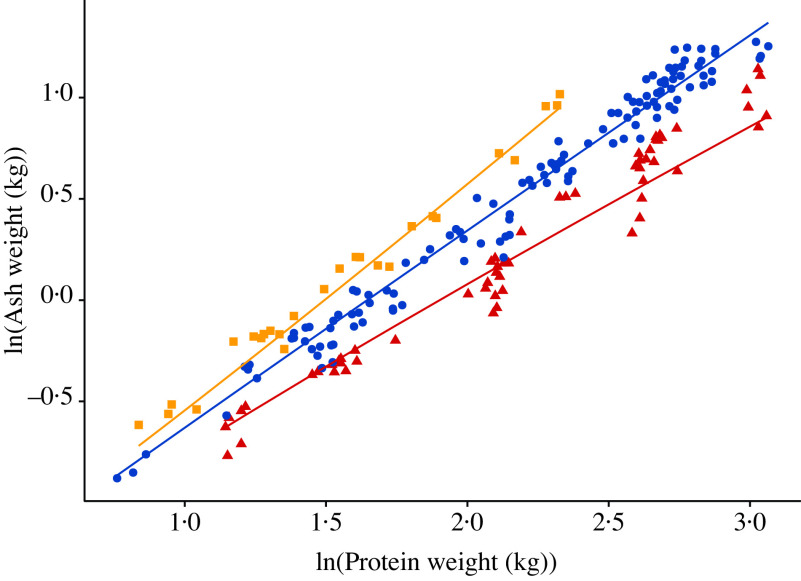
Relationship between body ash weight and body protein weight at the time of slaughter weights in growing and finishing pigs; individual data points (*n* 211) represent mean values for each considered dietary treatment reported in twenty-five studies (see online Supplementary material for a list of included studies). The data were expressed on the natural logarithmic (ln) scale, under the following three *ad libitum* feeding schedules: (1) pigs were given protein-deficient feeds; (2) pigs were given nutritionally balanced feeds; (3) pigs were given phosphorus-deficient feeds. Data: 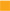, protein-deficient feed; 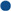, balanced feed; 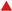, phosphorus-deficient feed.

The relationship between body ash (kg) and protein (kg) weights at the time of slaughter from the included literature data (see online Supplementary material for a list of included studies) is visualised in Fig. 4. A summary of the weighted least squares regression of ash weight (kg) on protein weight (kg) for pigs given: (1) nutritionally balanced feeds; (2) protein-deficient feeds and (3) P-deficient feeds is given in Table 3. The estimated allometric exponent *b* was: (1) 0·981 (95 % CI 0·951, 1·01) for pigs given balanced feeds; (2) 1·08 (95 % CI 1·02, 1·14) for pigs supplied with protein-deficient feeds; (3) 0·873 (95 % CI 0·821, 0·926) for pigs fed P-deficient feeds. Hence, the ash–protein isometry hypothesis could not be rejected for pigs given balanced feeds (*P* > 0·05), but was rejected in favour of allometric scaling for pigs fed protein-deficient feeds (*b* > 1; *P* < 0·01) and P-deficient feeds (*b* < 1; *P* < 0·001).

**Table 3.** Regression estimates, standard errors, 95 % confidence intervals and coefficients of determination (*R*^2^) for the fitted relationship: 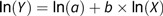, where *Y* was the body ash weight and *X* was the body protein weight under the following three *ad libitum* feeding schedules: (1) pigs were given protein-deficient feeds; (2) pigs were given nutritionally balanced feeds; (3) pigs were given phosphorus-deficient feeds* (Regression estimates, standard errors, 95 % confidence intervals and coefficients of determination)

Parameter	Feed class	Estimate	se	95 % CI	*P*	*R*^2^
ln(a)	Protein-deficient	−1·57	0·0477	−1·67, −1·48		0·983 (protein-deficient)
	Balanced	−1·63	0·0324	−1·70, −1·57		
	P-deficient	−1·68	0·0563	−1·79, −1·57		0·972 (balanced)
b	Protein-deficient	1·08	0·0289	1·02, 1·14	0·00880	
	Balanced	0·981	0·0150	0·951, 1·01	0·204	0·956 (P-deficient)
	P-deficient	0·873	0·0261	0·821, 0·926	1·18 × 10^–5^	

* Probability values were given for the direct test of whether the estimated relationship was isometric (*b* = 1). A total of 211 individual data points representing mean values for each considered dietary treatment reported in twenty-five studies (see online Supplementary material for a list of included studies) were used in the analysis.

High *R*^2^ values (0·983, 0·972 and 0·956 for protein-deficient, balanced and P-deficient feeds, respectively) indicated a good fit to the data. Fitting weighted least squares using the two variables in reversed roles (e.g. ash as the *x*-axis variable) or using reduced major axis regression of ash weight on protein weight produced similar outputs, indicating that the above results are robust to the assumptions of the regression models (online Supplementary material).

### Phosphorus partitioning in body pools – bones

**Fig. 5.**
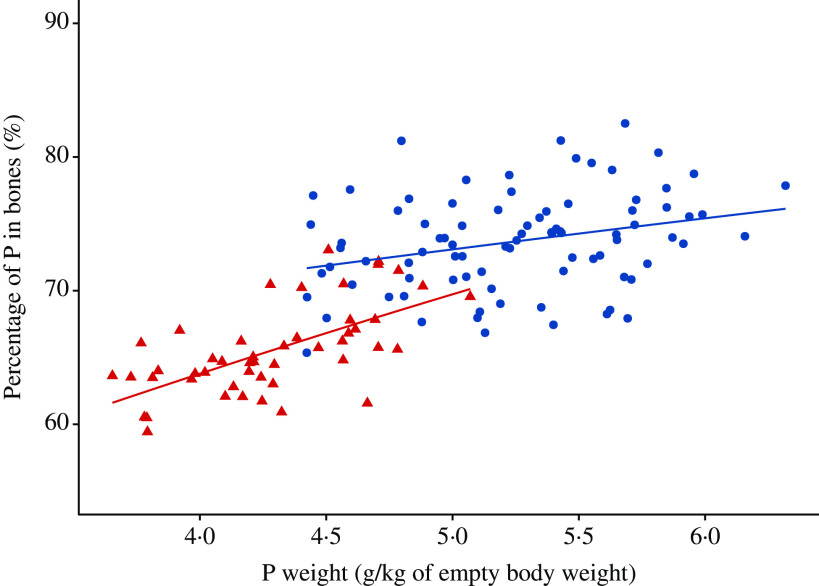
Estimated percentage of the body phosphorus weight in bones against scaled phosphorus weight (g/kg of empty body weight) in pigs at the time of slaughter. Individual data points (*n* 136) represent mean values for each considered dietary treatment reported in eleven studies (see online Supplementary material for a list of included studies), under the following two *ad libitum* feeding schedules: (1) pigs were given nutritionally balanced feeds; (2) pigs were given phosphorus-deficient feeds. Data: 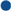, balanced feed; 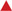, phosphorus-deficient feed.

The estimated percentage of bone P (P_bone%_) in the whole-body P of pigs at the time of slaughter from the reported literature data (see online Supplementary material for a list of included studies) is given in Fig. 5. A summary of the fitted beta regression model of P_bone%_ (%) on P (g/kg of eBW) for the two considered feeding schedules is given in Table 4. On the basis of the regression, P_bone%_ increased with increasing body P (*P* < 0·01 balanced feeds; *P* < 0·001 P-deficient feeds), rejecting the proposition that P_bone%_ in the body is constant. The slopes of the regression lines differed between the feeding schedules, with a smaller slope for the balanced feed group (balanced feeds, 95 % CI 0·0413, 0·204; P-deficient feeds, 95 % CI 0·209, 0·330). Dispersion was constant in the two models and did not depend on P (*P* > 0·05). The pseudo *R*^2^ values of 0·496 and 0·721 for the balanced feed and P-deficient feeds, respectively, indicated a moderate fit to the data.

**Table 4.** Regression estimates, standard errors, 95 % confidence intervals, squared correlation of linear predictor and link-transformed response (*R*^2^)^(62)^ and probability values in the final fitted beta regression models for estimated percentage of bone phosphorus in the whole-body phosphorus under the following two *ad libitum* feeding schedules: (1) pigs were given nutritionally balanced feeds; (2) pigs were given phosphorus-deficient feeds* (Regression estimates, standard errors, 95 % confidence intervals, squared correlation of linear predictor and link-transformed response and probability values)

Parameter	Feed class	Estimate	se	95 % CI	*P*	*R*^2^
Mean model						
Intercept	Balanced	0·390	0·232	−0·0408, 0·814	0·0925	0·496 (balanced)
	P-deficient	−0·507	0·212	−0·892, −0·114	0·0167	
Slope	Balanced	0·122	0·0441	0·0413, 0·204	0·00570	
	P-deficient	0·268	0·0294	0·209, 0·330	5·7 × 10^−8^	
Precision model						
Intercept	Balanced	150	22·9	100, 200	4·87 × 10^−11^	0·721 (P-deficient)
	P-deficient	318	64·1	142, 484	7·16 × 10^−7^	

* A total of 136 individual data points representing mean values for each considered dietary treatment reported in eleven studies (see online Supplementary material for a list of included studies) were used in the analysis.

### Model exploration

**Fig. 6.**
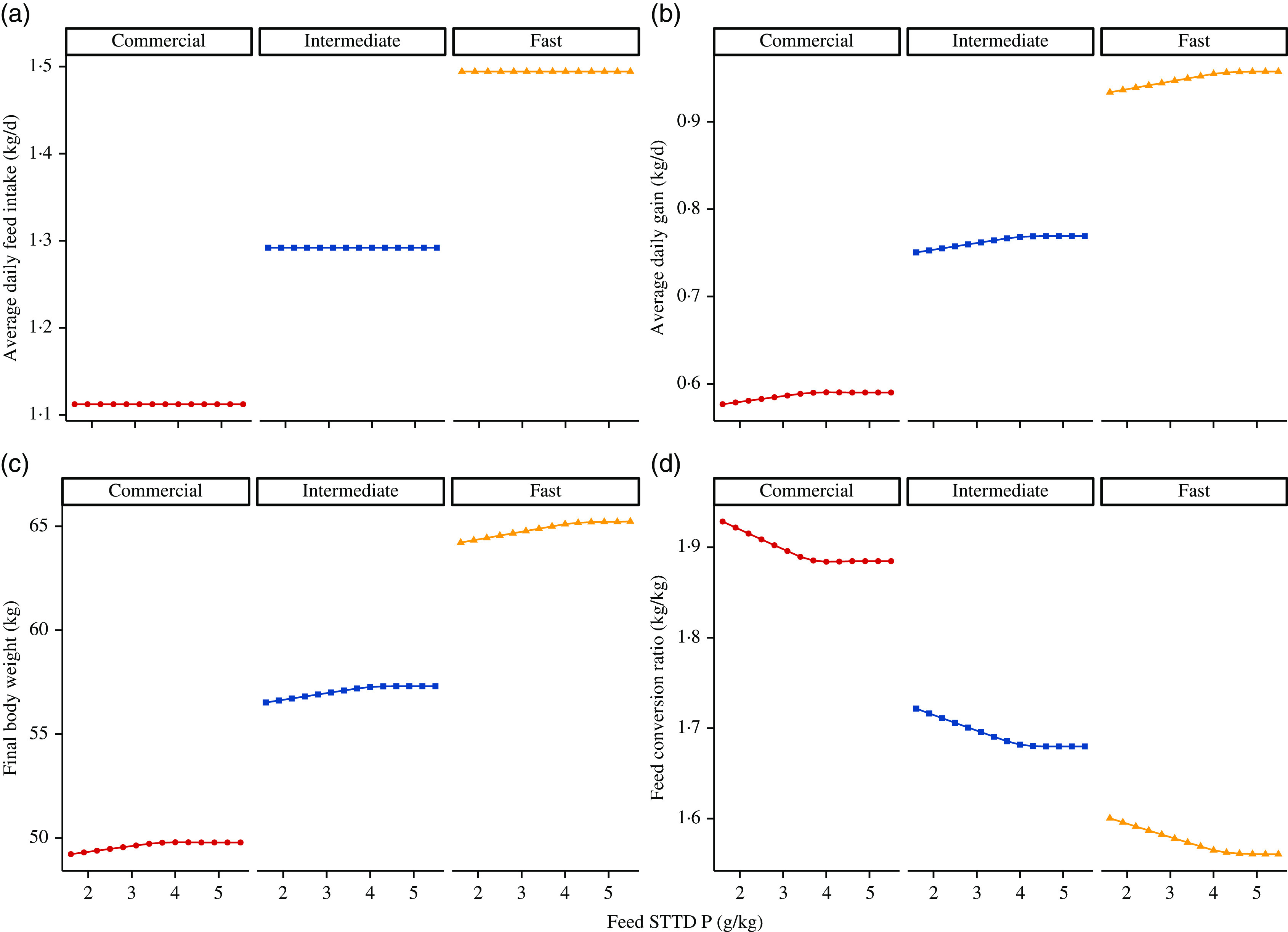
Simulated effects of feed standardised total tract digestible (STTD) phosphorus content (g/kg) on: (a) average daily feed intake (kg/d), (b) average daily gain (kg/d), (c) final body weight (kg) and (d) average feed conversion ratio (kg/kg) in pigs of three pig phenotypes^(34)^ growing from 25·0 kg live body weight. The three phenotypes were: (i) fast growing, 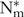
= 50·0 kg, L_m_ = 55·0 kg, B = 0·0125; (ii) intermediate growing, 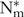 = 40·0 kg, L_m_ = 48·0 kg, B = 0·0118; and (iii) commercial, 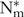 = 30·0 kg, L_m_ = 39·0 kg, B = 0·0110. Feeds were isoenergetic and contained STTD phosphorus contents ranging from 50 to 180 % of the current National Research Council guidelines^(11)^, which were supplied on an *ad libitum* basis for 42 d.

**Fig. 7.**
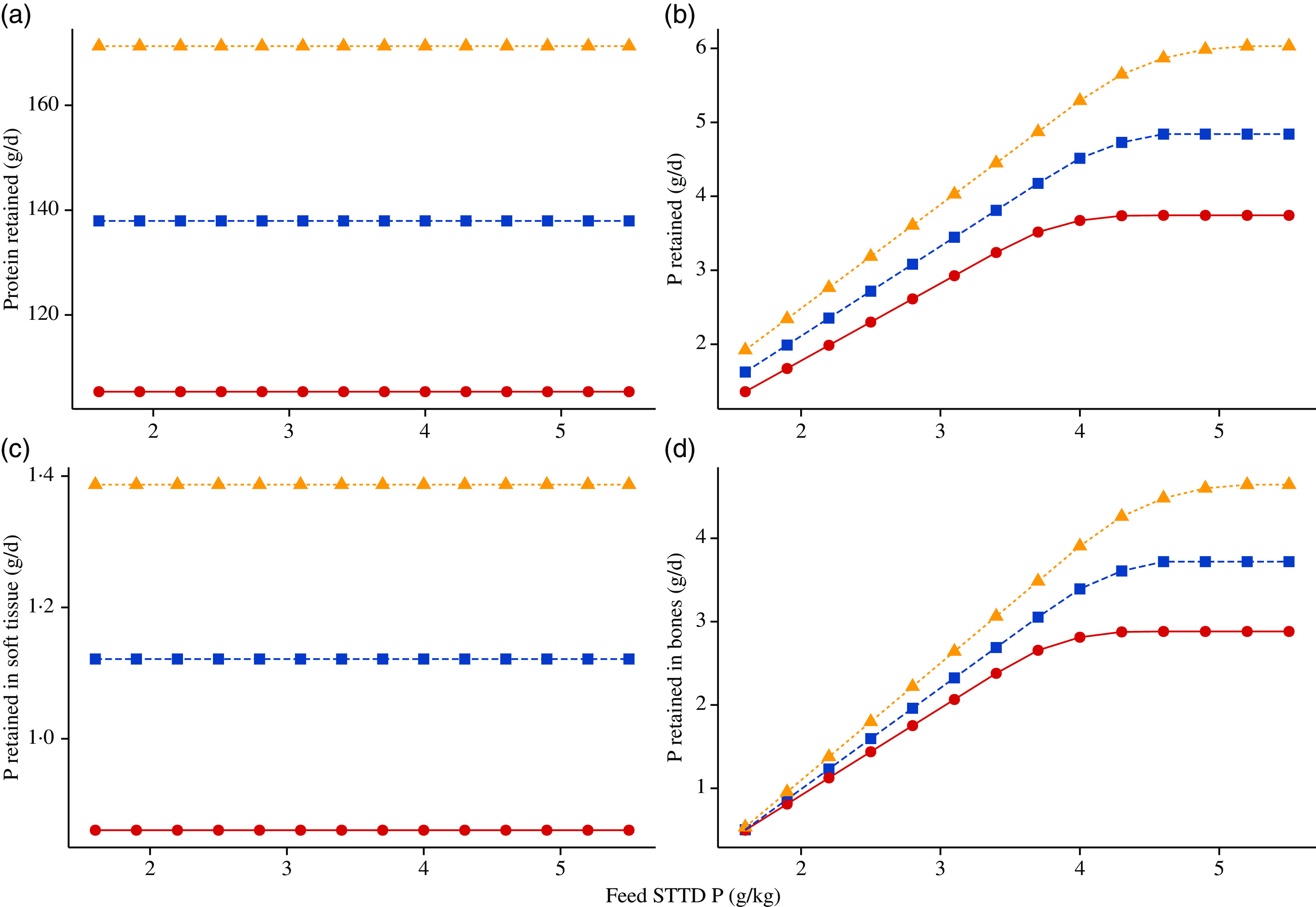
Simulated effects of feed standardised total tract digestible (STTD) phosphorus content (g/kg) on: (a) daily protein retention (g/d); (b) daily body phosphorus retention (g/d); (c) daily phosphorus retention in soft tissue (g/d); (d) daily phosphorus retention in bones (g/d) in pigs of three pig phenotypes^(34)^ growing from 25·0 kg live body weight. The three phenotypes were: (i) fast growing, 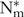 = 50·0 kg, L_m_ = 55·0 kg, B = 0·0125; (ii) intermediate growing, 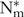 = 40·0 kg, L_m_ = 48·0 kg, B = 0·0118; and (iii) commercial, 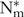 = 30·0 kg, L_m_ = 39·0 kg, B = 0·0110. Feeds were isoenergetic and contained STTD phosphorus contents ranging from 50 to 180 % of the current National Research Council guidelines^(11)^, which were supplied on an *ad libitum* basis for 42 d. Growth: 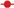, commercial; 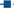, intermediate; 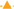, fast.

Simulated effects of different STTD P feed contents on ADFI (kg/d), ADG (kg/d), final BW (kg) and feed conversion ratio (kg/kg) for the three considered phenotypes (section Feed) are given in Fig. 6. For all three pig lines, the predicted ADFI remained unchanged with increasing STTD P feed content. The final BW increased with increasing STTD P feed content, which was attributed to an increase in P and ash retentions. Correspondingly, there was a decrease in the feed conversion ratio (increase in efficiency) and an increase in the ADG as STTD P feed content increased (%ADG increase from the lowest to the highest STTD P feed content: (1) commercial pig: 2·20 % and (2) intermediate growth type pig: 2·47 %; fast growth type pig: 2·51 %). Simulated daily retention of protein (g/d), P (g/d), P_soft_ (g/d) and P_bone_ (g/d) at different STTD P feed contents for the three considered phenotypes is summarised in Fig. 7. Within the considered range of STTD P feed contents, the daily protein retention remained constant, while the daily P retention increased with increasing STTD P feed content. The simulated P–protein relationship in the body conformed to the empirical evidence indicating that the P–protein relationship in the body is not constant and is dependent upon feed composition (section Relationship between ash–protein and phosphorus–protein body weights). Reductions in the STTD P feed content did not affect P_soft_ retention, but reduced P_bone_ retention because the mechanistic model allocated absorbed P intake to P_bone_ only after maintenance and P_soft_ retention was satisfied. Daily P_bone_ retention was 88, 86 and 82 % lower at the lowest than at the highest STTD P feed content for the fast-growth, intermediate-growth and commercial pig phenotypes, respectively.

### Model validation

**Fig. 8.**
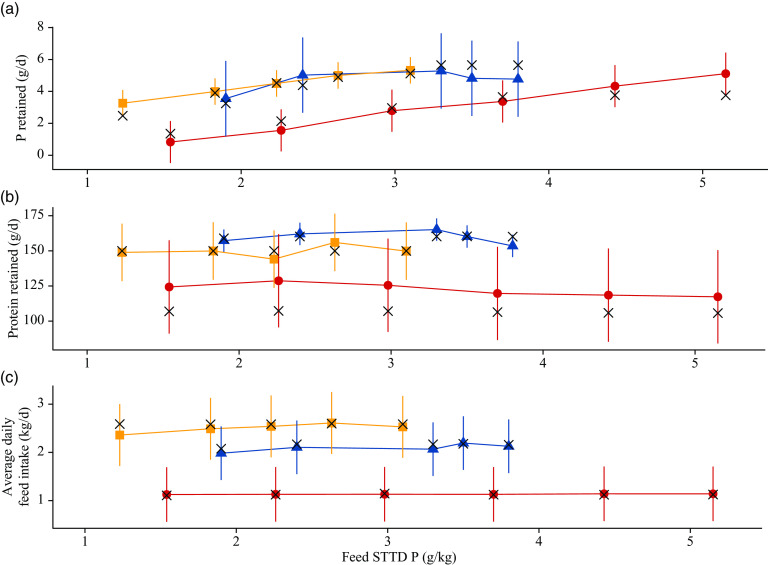
Model validation through a comparison of the simulated predictions from the mechanistic model^(76)^ for: (a) daily phosphorus retention (g/d); (b) daily protein retention (g/d); (c) average daily feed intake (kg/d) against the reported data (*n* 16 data points) originating from the following three papers: (1) 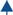, Adeola *et al.*^(78)^; (2) 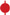, Pomar *et al.*^(77)^; and (3) 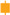, Ekpe *et al.*^(76)^. Predictions × data (means and 2 sd).

**Fig. 9.**
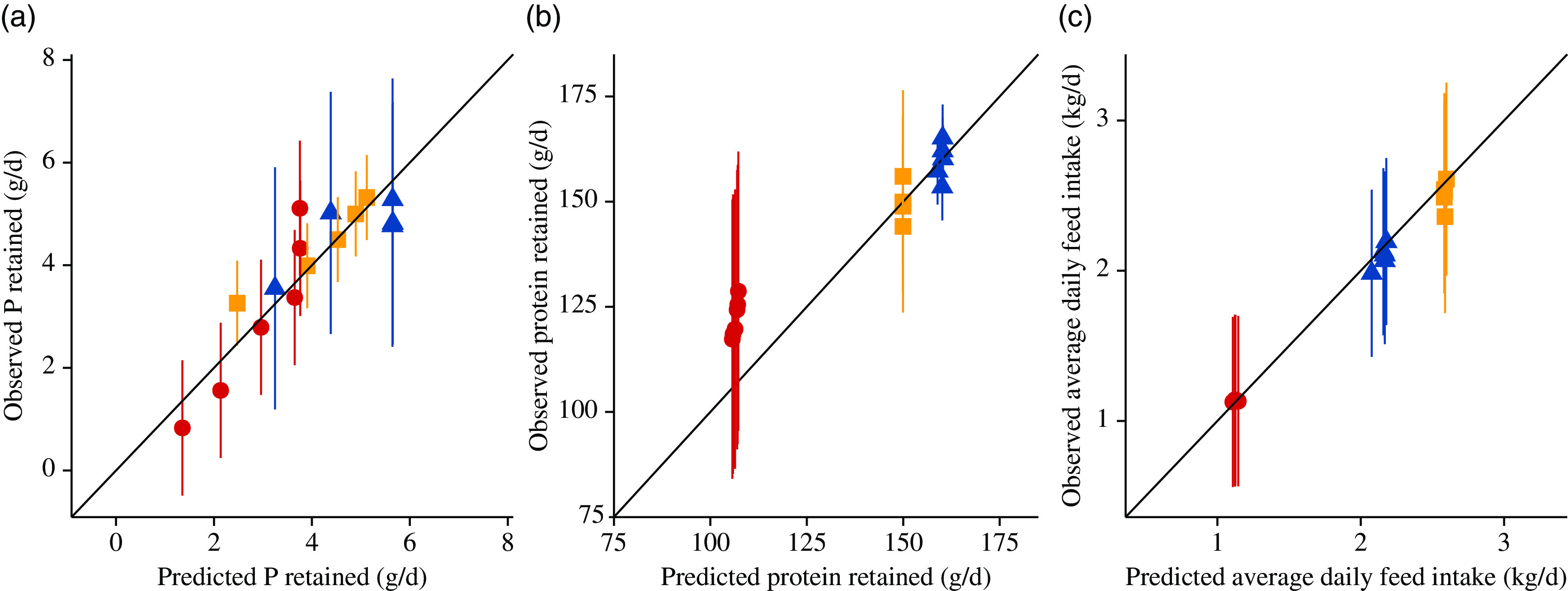
Pooled model validation: observed *v*. fitted values (generated by a mechanistic model). for: (a) daily phosphorus retention (g/d); (b) daily protein retention (g/d); (c) average daily feed intake (kg/d). Data originated (*n* 16) from the following three papers: (1) 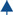, Adeola *et al.*^(78)^; (2) 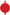, Pomar *et al.*^(77)^ and (3) 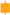, Ekpe *et al.*^(76)^. Values are represented as means and 2 sd.

The characteristics of the studies used for model validation are summarised in Table 5. Results of the model validation are presented in Table 6 and Figs. 8 and 9. The predicted daily P retention closely matched the observed data (Figs. 8 and 9), with all model predictions being inside the reported mean and 2sd. Predicted daily protein retention remained largely constant across all STTD P feed content. While daily protein retention predictions for the pig genotypes of Ekpe *et al.*^(76)^ and Pomar *et al.*^(77)^ were very close to the reported mean values, the simulated daily protein retention for the pig genotypes of Adeola *et al.*^(78)^ was systematically lower than the mean values (Figs. 8 and 9). All predictions of ADFI were very close to the reported mean values (Figs. 8 and 9). The scaled residuals against the predicted values did not reveal any systematic deviations across studies for the daily P retention, ADFI, but the error generally increased with increasing daily protein retention (online Supplementary material). Values of MAPE and *R*^2^ are given in Table 6. *R*^2^ ranged from 0·611 for the daily protein retention to 0·983 for the ADFI, while the MAPE ranged from 2·47 % for the ADFI to 15·7 % for the daily P retention.

**Table 5.** Characteristics of the included experiments used for model validation: (1) Adeola *et al.*^(78)^; (2) Pomar *et al.*^(77)^; and (3) Ekpe *et al.*^(76)^

	Adeola *et al.*^(78)^	Pomar *et al.*^(77)^	Ekpe *et al.*^(76)^
Initial BW (kg)	24·7–25·6	16·3	50·9–56·6
Final BW (kg)	34·3–33·5	98·8–106·2	57·3–64·2
Number of pigs per dietary treatment	8	8–10	4
Trial length (d)	14	87	8
Housing type	Individually housed	Individually housed	Individually housed
ME feed content (MJ/kg of feed)	13·5–13·9	13·7–13·8	13·1
Crude protein feed content (g/kg of feed)	183–184	153–169	155–158
STTD P feed content (g/kg of feed)	1·54–5·15	1·23–3·1	1·91–3·84
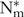 (kg)	28·7*	21·9*	27·2*
B	0·0125*	0·0212*	0·0158*
L_m_ (kg)	53·6*	57·8*	40·9*

BW, body weight; ME, metabolisable energy; STTD P, standardised total tract digestible P; 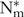
protein weight at maturity; B, daily growth rate; L_m_, lipid at maturity.

* Estimations of values not reported.

**Table 6.** Mean absolute percentage errors (MAPE) and coefficients of determination (*R*^2^) of mechanistic model predictions for: (1) daily phosphorus retention (g/d); (2) daily protein retention (g/d); (3) average daily feed intake (kg/d)* (Mean absolute percentage errors and coefficients of determination)

Predicted variable	MAPE (%)	*R*^2^
Daily P retention (g/d)	15·7	0·790
Daily protein retention (g/d)	5·95	0·611
Average daily feed intake (kg/d)	2·47	0·983

* Predictions corresponded to simulations from the three papers are summarised in Figs. 8 and 9, and Table 5. A total of sixteen individual data points representing mean values for each considered dietary treatment were used in the analysis.

## Discussion

### Do animals change their feed intake when given feeds with deficient phosphorus content?

On the basis of our statistical analysis of the relevant published literature data, we found no evidence that pigs given *ad libitum* access to P-deficient feeds modify their corresponding FI response. *A priori*, there were three main possibilities regarding the FI regulation in the context of P-deficient feeds: (1) decrease; (2) no change and (3) increase. Each of these alternatives has different consequences upon nutrient excretion, animal performance and welfare. A decrease in the FI could impair the growth. Moreover, it could lead to cases where pigs retain inadequate quantities of P, potentially weakening the skeletal structure and affecting many non-skeletal physiological processes, which require P^(36,40)^. While no change in the FI response could lead to satisfactory performance (in terms of ADG) and to reductions in P excretion in the short-term, it could impact body composition or even pose health and welfare challenges through, for example, inadequate bone mineralisation^(14–20)^. A FI increase could result in a fatter animal, impacting the carcass value and would raise excretion of all the other resources consumed in excess.

Empirically, the aforementioned FI responses have a considerable literature support. While a decrease in FI, accompanied by reductions in the growth performance, was reported in piglets and growing-finishing pigs^(79–82)^, other researchers found no changes in FI in the context of pigs of all stages of production^(83–85)^, broilers^(86–88)^ and laying hens^(89)^. In laboratory animals, provision of P-deficient feeds was reported to lead to an increase in the overall FI^(90,91)^; this could be an attempt to satisfy maintenance and growth requirements of the most limiting nutrient^(63,92,93)^, which would be consistent with FI responses in pigs given energy^(94)^ and protein-deficient feeds^(41,95–97)^.

Contextualising our findings in light of the aforementioned additional empirical evidence is challenging, since the magnitude, the duration and the timing during growth of these mineral deficiencies could be expected to influence the FI response^(98)^. Moreover, since animals possess large reservoirs of P, largely located in bones^(99)^, which can be mobilised if necessary, the presence of these reserves could have an impact on the observed FI response. For example, pigs given feeds that restrict P bone retention but not the maintenance functions and lean tissue retention could attempt to modify their FI only once the depletion of P bone reserves reaches a threshold level. Hence, it may be that the growth of lean tissue could be supported by P resorption from the bone tissues and that a different FI response (e.g. a FI reduction as observed in Baker *et al.*^(100)^ under the magnitude of P deficiency perceived to be higher than other studies included in the database) would occur if lean tissues were compromised by the P-deficient intake.

From an *in silico* perspective, the relevant models of P utilisation in livestock animals typically use FI data as a model input or estimate the FI based on other traits^(22–25,101,102)^. To date, only the Symeou *et al.*^(21)^ model of P utilisation predicts different FI in cases of P-deficient feeds; this model assumes that FI is controlled by the energy and protein requirements and is largely independent of the P feed supply. In the context of other pig growth models, FI is estimated by relating it to BW^(11,26)^ or by assuming that animals attempt to eat for the most limiting nutrient resource^(27,30,103,104)^. Our present findings would give support to models that assume independency between P feed supply and FI^(11,21,26)^, at least while bone P can be resorbed for maintenance and growth.

### How is the relationship between bone mineralisation and muscle tissue affected when animals are given feeds with deficient phosphorus content?

Our data-based results indicate that when pigs are given access to balanced feeds, there is an isometric relationship between ash and protein, and between P and protein, which is consistent with the literature^(21,27)^. This result means that the proportionality between muscles and load bearing bones is preserved when feeds are non-limiting. Contrastingly, our results indicate that under the conditions of nutritional deficiency, there is an allometric relationship between ash and protein, and between P and protein. This means that, when given protein-deficient feeds, the animal will grow more ash and P in relation to body protein, and when given P-deficient feeds, the animal will grow more body protein in relation to ash and P. These empirical results are contrary to the common modelling assumption that mineral–protein relationships are isometric^(105)^ across feed composition scenarios, which was utilised in the model of P retention by Symeou *et al.*^(21)^. Hence, caution should be exercised when using this assumption in the context of nutrient-deficient feeds, as it may yield inaccurate estimates of intake requirements and growth and body composition under such nutrient deficiency conditions.

### Are the phosphorus intake resources allocated differently within the body when animals are given feeds with deficient phosphorus content?

Our findings support the concept of prioritisation when there are limited absorbed P resources, that is, P retention in soft tissue is prioritised over P retention in bones. This result indicates that the animal will attempt to maintain its maximum lean tissue retention because the bones could tolerate reduced P levels. This preferential allocation of limited P resources conforms with the above findings that the P–protein and ash–protein relationships are allometric for nutritionally deficient feeds. Moreover, this prioritisation of P accretion in soft tissue is consistent with the Létourneau-Montminy *et al.*^(22)^ model of P dynamics and with the NRC^(11)^ recommendation that P requirements to sustain maximum rate and efficiency of weight gain are at least 15 % lower than those for maximum bone strength and bone density, although it is not assumed in the Symeou *et al.*^(21)^ model.

### Model results and validation

Using our data-supported answers to Q1–Q3, a dynamic, mechanistic model was developed to explore the consequences of these findings. The model describes pig growth, body tissue and skeleton composition across scenarios where digestible P feed content varies from suitable to deficient. In developing the model, we revised and updated the assumptions of the current mechanistic models of pig growth pertaining dynamic P composition^(21,22)^.

We considered reductions in P feed content from 180 % to 50 % of the current NRC guidelines for 25–50 kg pigs^(11)^. The simulations illustrated how these reductions affected P retention in the bones but not P retention in the soft tissue for three pig phenotypes. Simultaneously, protein retention was maintained, while ash retention was reduced with decreasing P feed content. Hence, lowering P feed content had a marginal effect on performance characteristics such as ADG and feed conversion ratio. Therefore, based on our simulations, recommendations by NRC^(11)^ for reducing P feeding seem plausible if ADG is the chosen response criterion. However, care should be taken to ensure that P retention in bones is not compromised to a degree that could pose animal health and welfare risks.

The suitability of the mechanistic model based on the data-supported assumption was tested by assessing its predictions against independent published data, that is, data that were not included in the meta-regressions for addressing Q1–Q3. The studies used for validation were chosen at random among all identified studies with *ad libitum* dietary treatments after grouping them in terms of P feed content, ranging from deficient to abundant. It should also be noted that it was necessary to estimate the pig phenotype parameters required to run the mechanistic model (section Estimation of pig phenotype parameters) before generating simulated predictions of the variables of interest. As a result, these predictions are to some degree linked to these parameter estimates and hence are not fully independent of the observations used for their validation. Predictions of ADFI were more accurate than those of protein and P retentions (mean absolute percentage errors of 2·47 *v*. 5·95 and 15·7 %, respectively) and could be attributed to challenges in the estimation of digestibility and efficiency of utilisation for protein and P. There was no systematic error in the predictions of P retention, ADFI, but protein retention was consistently underestimated for one study^(78)^. Potential reasons for this underestimation could be due to inaccuracies in the estimated pig phenotype parameters or in the reported feed composition, which were used as a model input, or could be due to the aforementioned uncertainties concerning the digestibility and efficiency of utilisation values for protein and P.

### Model limitations

A major limitation of the present model and other mechanistic models of P dynamics^(21,22)^ is their inability to account for the effects of nutritional history on the subsequent growth performance. Consequently, our model is unable to simulate scenarios where a pig is allowed to transition from one feed to another, most notably when this change occurs from P deficient to balanced diets. Such scenarios are progressively more likely to occur in commercial settings, where the number of feeding phases is increasing in an attempt to formulate feeds that more closely match the dynamically changing nutritional requirements of the animals^(106)^. A phase of accelerated growth has been demonstrated to occur in pigs when favourable conditions are restored following a period of protein deficiency^(107–113)^ or energy/feed restrictions^(114–117)^. Evidence for such a compensatory growth in the context of mineral nutrition is limited and inconclusive^(19,118)^, but could be an area of future research.

Another limitation of the model relates to the current assumption that the efficiency of utilisation of dietary P is independent of the feed composition and body size^(21)^. Literature on this subject is inconclusive; while reductions in P feed content have been demonstrated to have no effect on P utilisation in piglets^(119)^, contrary findings were reported by Varley *et al.*^(120)^, where P utilisation increased as P feed content was reduced. Similarly, the effect of body size on P utilisation is unknown; for example, Pettey *et al.*^(121)^ reported that the efficiency of P utilisation decreases with increasing BW, while the converse was reported by Kemme *et al.*^(122)^. More research is required to elucidate the correct functional relationship between P utilisation and feed composition and size of the animal.

### Wider applications and further work

Our findings are likely to be relevant to feed formulation and the estimation of P requirements in other monogastric species, especially broiler chickens. Moreover, since mathematical models applied in these livestock species share many common concepts with models of pig growth^(104,123)^, the assumptions highlighted in our paper merit similar revisions for these species.

Due to contrasting literature findings concerning the FI response in pigs given P-deficient feeds, future research should involve experiments that thoroughly investigate how the duration of P-deficiency impacts this response.

Our results on the relationships between body P and protein and between body ash and protein under different nutritional scenarios were restricted by the amount of information available in the peer-reviewed literature. Specifically, the data on P and protein BW were limited, with a particular lack of data on pigs given access to deficient feeds, which meant it was not possible to evaluate the P–protein relationship within the body for animals supplied with protein-deficient feeds. While the present results could seem consistent with the idea that muscle development follows rather than precedes skeletal development, further *in vivo* experiments are required to generate additional data needed to fully capture the dynamics between different body components in pigs kept under different nutritional scenarios.

In addition, owing to a lack of data, it was not possible to validate the model predictions of the separate P retention in soft tissue and in bones. This limitation highlights a need for further *in vivo* experiments on the P dynamics within these two pools in the body. Moreover, generating such data would help with our current, limited understanding of the role of P bone reserves in FI regulation and growth and could help to increase the predictive capabilities of future models in the context of severe P deficiencies, that is, in circumstances when the animal is unable to draw upon its skeletal reserves.

## Conclusions

Our paper provides answers to central questions in the nutritional theory of monogastric species, that is, how intake is regulated given feeds of different and possibly deficient nutrient composition, and how deficient nutrient resources are partitioned within the body.

We assessed assumptions about the processes associated with these questions that are currently incorporated in mechanistic models of P utilisation in growing pigs. Specifically, we found no evidence to suggest that pigs attempt to respond to P-deficient feeds by modifying their FI. Secondly, we demonstrated that the common assumptions of isometry between protein and P, and between protein and a share supported only for animals given nutritionally balanced feeds. Lastly, we provided additional evidence for prioritisation of P retention in soft tissue when this nutrient is deficient within the body.

Our modelling study built on the data-supported assumptions illustrated the effects of P deficiencies on performance and body composition of growing pigs. A better understanding of the consequences of reducing P feed contents should lead to a decline in the use of oversupply as a safety margin in the present P feeding guidelines and should support efforts to reduce P excretion from commercial pig production systems.

## Supplementary material

For supplementary material accompanying this paper visit http://dx.doi.org/10.1017/S0007114520000975. click here to view supplementary material
