# Supplementary material for: How do pigs deal with dietary phosphorus deficiency?
Source: Br J Nutr. 2020 Mar 16;124(3):256–72. doi: 10.1017/S0007114520000975 (PMC7512807; doi:10.1017/S0007114520000975)
Supplement: Supplementary file 1 [file S0007114520000975sup001.docx]

**Supplementary Material A:** List of studies included in the feed intake regulation analysis

1. Saraiva A, Donzele JL, Oliveira RFMd *et al.* (2011) Available phosphorus levels in diets for 30 to 60 kg female pigs selected for meat deposition by maintaining calcium and available phosphorus ratio. *R Bras Zootec* 40, 587-592.

2. Saraiva A, Donzele JL, Oliveira RFMd *et al.* (2009) Available phosphorus levels in diets for swine from 15 to 30 kg genetically selected for meat deposition. *R Bras Zootec* 38, 307-313.

3. Baker S, Kim B, Stein H (2013) Comparison of values for standardized total tract digestibility and relative bioavailability of phosphorus in dicalcium phosphate and distillers dried grains with solubles fed to growing pigs. *J Anim Sci* 91, 203-210.

4. Arouca CLC, Silva FCdO, Fontes DdO *et al.* (2012) Available phosphorus levels for 95 to 120 kg barrows genetically selected for lean gain. *R Bras Zootec* 41, 1433-1441.

5. Saraiva A, Donzele J, Oliveira R *et al.* (2012) Phosphorus requirements for 60-to 100-kg pigs selected for high lean deposition under different thermal environments. *J Anim Sci* 90, 1499-1505.

6. Zhai H, Adeola O (2015) True digestible phosphorus requirement for forty-to eighty-kilogram pigs. *J Anim Sci* 93, 5711-5717.

7. Zhai H, Adeola O (2013) True digestible phosphorus requirement of 10-to 20-kg pigs. *J Anim Sci* 91, 3716-3723.

8. Zhai H, Adeola O (2013) True digestible phosphorus requirement for twenty-to forty-kilogram pigs. *J Anim Sci* 91, 5307-5313.

9. Alebrante L, Donzele JL, Oliveira RFMd *et al.* (2011) Available phosphorus for 15-to 30-kg pigs kept in hot environment. *R Bras Zootec* 40, 2725-2731.

10. Bünzen S, Rostagno HS, Kiefer C *et al.* (2012) Níveis de fósforo digestível para suínos em fase de crescimento. *R Bras Zootec* 41, 320-325.

11. Hastad C, Dritz S, Tokach M *et al.* (2004) Phosphorus requirements of growing-finishing pigs reared in a commercial environment. *J Anim Sci* 82, 2945-2952.

12. Arouca CLC, Silva FCdO, Fontes DdO *et al.* (2012) Available phosphorus in diets for 15 to 30 kg pigs genetically selected for meat deposition. *R Bras Zootec* 41, 65-71.

13. Campos PF, Silva FCdO, Ferreira AS *et al.* (2012) Available phosphorus in diets with or without ractopamine for late finishing gilts. *R Bras Zootec* 41, 630-635.

14. Mavromichalis I, Hancock J, Kim I *et al.* (1999) Effects of omitting vitamin and trace mineral premixes and (or) reducing inorganic phosphorus additions on growth performance, carcass characteristics, and muscle quality in finishing pigs. *J Anim Sci* 77, 2700-2708.

15. Carter S, Cromwell G (1998) Influence of porcine somatotropin on the phosphorus requirement of finishing pigs: I. Performance and bone characteristics. *J Anim Sci* 76, 584-595.

**Supplementary Material B:** List of studies included in the analysis of the relationship between mineral and protein weights

**Phosphorus-protein relationship**

1. Bikker P, Dekker R, van Diepen JTM *et al.* (2013) *Behoefte en vastlegging van fosfor bij vleesvarkens*. Wageningen UR Livestock Research.

2. Columbus D, Niven S, Zhu C *et al.* (2010) Phosphorus utilization in starter pigs fed high-moisture corn-based liquid diets steeped with phytase. *J Anim Sci* 88, 3964-3976.

3. Fandrejewski H, Rymarz A (1986) Effect of feeding level on Ca, P, K and Na content in the bodies of growing boars and gilts. *Livest Prod Sci* 14, 211-215.

4. Fandrejewski H, Weremko D, Raj S *et al.* (1999) Performance, body and carcass composition and bone characteristics of pigs fed rapeseed and soyabean meal-cereal diets supplemented with microbial phytase. *J Anim Feed Sci* 8, 533-547.

5. Hendriks W, Moughan P (1993) Whole-body mineral composition of entire male and female pigs depositing protein at maximal rates. *Livest Prod Sci* 33, 161-170.

6. Jongbloed AW (1987) Phosphorus in the feeding of pigs: effect of diet on the absorption and retention of phosphorus by growing pigs, Instituut voor Veevoedingsonderzoek (I.V.V.O.) Lelystad.

7. Mahan D, Shields Jr R (1998) Macro-and micromineral composition of pigs from birth to 145 kilograms of body weight. *J Anim Sci* 76, 506-512.

8. Rousseau Xr (2013) Optimisation de l'utilisation de phosphore alimentaire chez le porc et le poulet en croissance, Université de Tours.

9. Rymarz A, Fandrejewski H, Kielanowski J (1982) Content and retention of calcium, phosphorus, potassium and sodium in the bodies of growing gilts. *Livest Prod Sci* 9, 399-407.

10. Zomeño C, Gispert M, Carabús A *et al.* (2016) Predicting the carcass chemical composition and describing its growth in live pigs of different sexes using computed tomographys. *Animal* 10, 172-181.

11. Nielsen AJ (1973) Anatomical and chemical composition of Danish Landrace pigs slaughtered at 90 kilograms live weight in relation to litter, sex and feed composition. *J Anim Sci* 36, 476-483.

**Ash-protein relationship**

1. Balfagón-Romeo A (2006) Nutritional Approach to Mineral Over-supplementation in Grow-finish Pigs: Organic Trace Minerals and Phosphorus Body Accretion, University of Kentucky.

2. Barea R, Nieto R, Lara L *et al.* (2006) Effects of dietary protein content and feeding level on carcass characteristics and organ weights of Iberian pigs growing between 50 and 100 kg live weight. *Animal Sci* **82**, 405-413.

3. Bikker P, Dekker R, van Diepen JTM *et al.* (2013) *Behoefte en vastlegging van fosfor bij vleesvarkens*. Wageningen UR Livestock Research.

4. Campbell R, Dunkin A (1983) The influence of protein nutrition in early life on growth and development of the pig: 1. Effects on growth performance and body composition. *Br J Nutr* **50**, 605-617.

5. Campbell RG, Biden RS (1978) The effect of protein nutrition between 5.5 and 20 kg live weight on the subsequent perfromance and carcass quality of pigs. *Anim Prod* **27**, 223-228.

6. Caperna T, Steele N, Komarek D *et al.* (1990) Influence of dietary protein and recombinant porcine somatotropin administration in young pigs: growth, body composition and hormone status. *J Anim Sci* **68**, 4243-4252.

7. Columbus D, Niven S, Zhu C *et al.* (2010) Phosphorus utilization in starter pigs fed high-moisture corn-based liquid diets steeped with phytase. *J Anim Sci* **88**, 3964-3976.

8. Conde-Aguilera JA, Aguinaga M, Aguilera J *et al.* (2011) Nutrient and energy retention in weaned Iberian piglets fed diets with different protein concentrations. *J Anim Sci* **89**, 754-763.

9. Fandrejewski H, Rymarz A (1986) Effect of feeding level on Ca, P, K and Na content in the bodies of growing boars and gilts. *Livest Prod Sci* **14**, 211-215.

10. Fandrejewski H, Weremko D, Raj S *et al.* (1999) Performance, body and carcass composition and bone characteristics of pigs fed rapeseed and soyabean meal-cereal diets supplemented with microbial phytase. *J Anim Feed Sci* **8**, 533-547.

11. Ferguson N, Gous R (1997) The influence of heat production on voluntary food intake in growing pigs given protein-deficient diets. *Animal Sci* **64**, 365-378.

12. Hendriks W, Moughan P (1993) Whole-body mineral composition of entire male and female pigs depositing protein at maximal rates. *Livest Prod Sci* **33**, 161-170.

13. Jongbloed AW (1987) Phosphorus in the feeding of pigs: effect of diet on the absorption and retention of phosphorus by growing pigs, Instituut voor Veevoedingsonderzoek (I.V.V.O.) Lelystad.

14. Kerr B, Southern L, Bidner T *et al.* (2003) Influence of dietary protein level, amino acid supplementation, and dietary energy levels on growing-finishing pig performance and carcass composition. *J Anim Sci* **81**, 3075-3087.

15. Kyriazakis I, Leus K, Emmans G *et al.* (1993) The effect of breed (Large White× Landrace ν. purebred Meishan) on the diets selected by pigs given a choice between two foods that differ in their crude protein contents. *Animal Sci* **56**, 121-128.

16. Mahan D, Shields Jr R (1998) Macro-and micromineral composition of pigs from birth to 145 kilograms of body weight. *J Anim Sci* **76**, 506-512.

17. Martinez-Ramirez H, Jeaurond E, de Lange C (2008) Dynamics of body protein deposition and changes in body composition after sudden changes in amino acid intake: I. Barrows. *J Anim Sci* **86**, 2156-2167.

18. Martinez-Ramirez H, Jeaurond E, De Lange C (2008) Dynamics of body protein deposition and changes in body composition after sudden changes in amino acid intake: II. Entire male pigs. *J Anim Sci* **86**, 2168-2179.

19. Moughan P, Smith W, Stevens E (1990) Allometric growth of chemical body components and several organs in the pig (20–90 kg liveweight). *New Zeal J Agr Res* **33**, 77-84.

20. Rousseau Xr (2013) Optimisation de l'utilisation de phosphore alimentaire chez le porc et le poulet en croissance, Université de Tours.

21. Rymarz A, Fandrejewski H, Kielanowski J (1982) Content and retention of calcium, phosphorus, potassium and sodium in the bodies of growing gilts. *Livest Prod Sci* **9**, 399-407.

22. Shields Jr R, Mahan D, Graham P (1983) Changes in swine body composition from birth to 145 kg. *J Anim Sci* **57**, 43-54.

23. Williams N, Stahly T, Zimmerman D (1997) Effect of chronic immune system activation on the rate, efficiency, and composition of growth and lysine needs of pigs fed from 6 to 27 kg. *J Anim Sci* **75**, 2463-2471.

24. Wyllie D, Speer V, Ewan R *et al.* (1969) Effects of starter protein level on performance and body composition of pigs. *J Anim Sci* **29**, 433-438.

25. Zomeño C, Gispert M, Carabús A *et al.* (2016) Predicting the carcass chemical composition and describing its growth in live pigs of different sexes using computed tomographys. *Animal* **10**, 172-181.

**Supplementary Material C:** List of studies included in the analysis of the allocation of Phosphorus resources - bones

1. Bikker P, Dekker R, van Diepen JTM *et al.* (2013) *Behoefte en vastlegging van fosfor bij vleesvarkens*. Wageningen UR Livestock Research.

2. Columbus D, Niven S, Zhu C *et al.* (2010) Phosphorus utilization in starter pigs fed high-moisture corn-based liquid diets steeped with phytase. *J Anim Sci* 88, 3964-3976.

3. Fandrejewski H, Rymarz A (1986) Effect of feeding level on Ca, P, K and Na content in the bodies of growing boars and gilts. *Livest Prod Sci* 14, 211-215.

4. Fandrejewski H, Weremko D, Raj S *et al.* (1999) Performance, body and carcass composition and bone characteristics of pigs fed rapeseed and soyabean meal-cereal diets supplemented with microbial phytase. *J Anim Feed Sci* 8, 533-547.

5. Hendriks W, Moughan P (1993) Whole-body mineral composition of entire male and female pigs depositing protein at maximal rates. *Livest Prod Sci* 33, 161-170.

6. Jongbloed AW (1987) Phosphorus in the feeding of pigs: effect of diet on the absorption and retention of phosphorus by growing pigs, Instituut voor Veevoedingsonderzoek (I.V.V.O.) Lelystad.

7. Mahan D, Shields Jr R (1998) Macro-and micromineral composition of pigs from birth to 145 kilograms of body weight. *J Anim Sci* 76, 506-512.

8. Rousseau Xr (2013) Optimisation de l'utilisation de phosphore alimentaire chez le porc et le poulet en croissance, Université de Tours.

9. Rymarz A, Fandrejewski H, Kielanowski J (1982) Content and retention of calcium, phosphorus, potassium and sodium in the bodies of growing gilts. *Livest Prod Sci* 9, 399-407.

10. Zomeño C, Gispert M, Carabús A *et al.* (2016) Predicting the carcass chemical composition and describing its growth in live pigs of different sexes using computed tomographys. *Animal* 10, 172-181.

11. Nielsen AJ (1973) Anatomical and chemical composition of Danish Landrace pigs slaughtered at 90 kilograms live weight in relation to litter, sex and feed composition. *J Anim Sci* 36, 476-483.

**Supplementary Material D:** Examples of diagnostic plots from the fitted regression models

**Phosphorus-protein relationship**

- QQ plot of residuals obtained from the weighted least squares regression with Phosphorus weight on protein weight (balanced feeds)


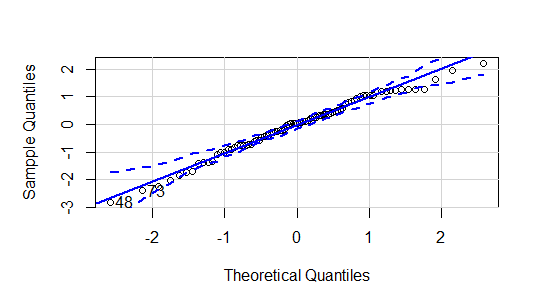


- Plot of residuals vs fitted values obtained from the weighted least squares regression with Phosphorus weight on protein weight (balanced feeds)


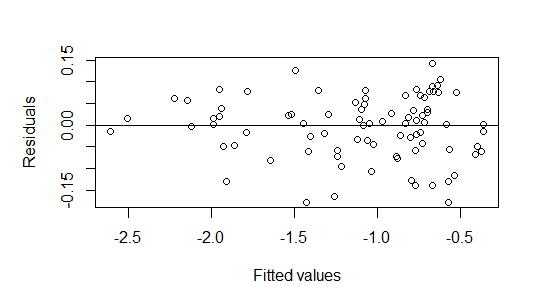


**Ash-protein relationship**

- QQ plot of residuals obtained from the weighted least squares regression with ash weight on protein weight (balanced feeds)


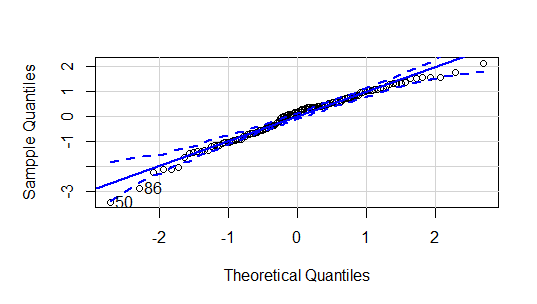


- Plot of residuals vs fitted values obtained from the weighted least squares regression with ash weight on protein weight (balanced feeds)


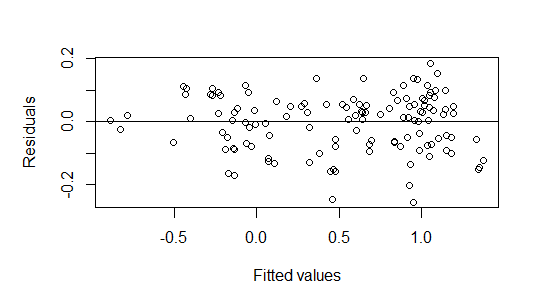


**Allocation of Phosphorus resources**

- Half normal plot of residuals obtained from the final beta regression model

**
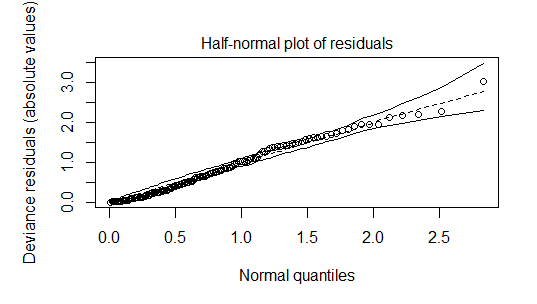
**

- Plot of residuals vs indices of the observations obtained from the final beta regression model

**
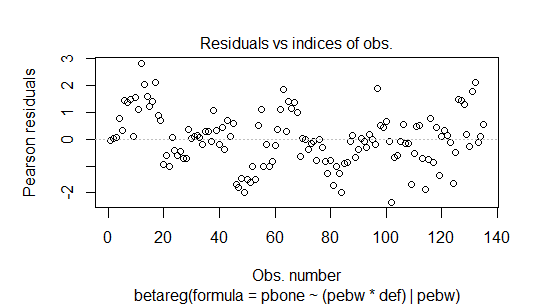
**

**Supplementary Material E:**

1) Average daily feed intake (ADFI; kg/d) against feed standardised total tract digestible Phosphorus (P) content (Feed STTD P content; g/kg): individual datapoints (n = 97) represent mean values for each considered dietary treatment reported in 15 studies

**
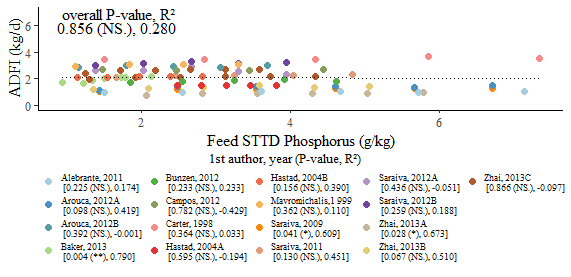
**

2) The metabolic bodyweight (BW^0.75^)-scaled average daily feed intake (ADFI; kg/kg/d) against feed standardised total tract digestible Phosphorus (P) content (Feed STTD P content; g/kg): individual datapoints (n = 97) represent mean values for each considered dietary treatment reported in 15 studies

**
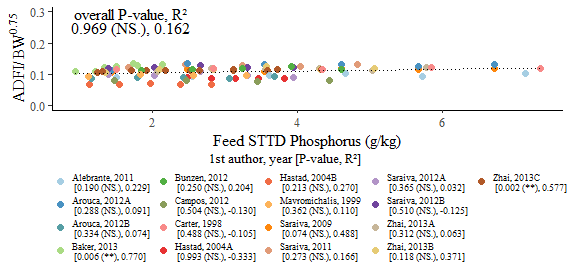
**

3) The metabolic bodyweight (BW^0.66^)-scaled average daily feed intake (ADFI; kg/kg/d) against feed standardised total tract digestible Phosphorus (P) content (Feed STTD P content; g/kg): individual datapoints (n = 97) represent mean values for each considered dietary treatment reported in 15 studies

**
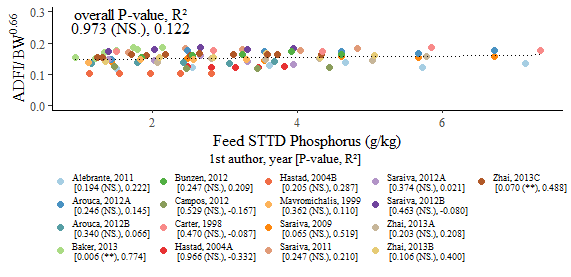
**

**Supplementary Material F:** An alternative analysis of the relationships between mineral and protein weight in pig body

**Phosphorus-protein relationship**

**Repeating model fitting using the two variables of interest in reversed roles (e.g. with Phosphorus (P) as the *x*-axis variable instead)**

| **Parameter** | **Feed class** | **Estimate** | **SE** | **95 % CI** | **Probability** | **R^2^** |
| --- | --- | --- | --- | --- | --- | --- |
| log(a) | Balanced | 3.39 | 0.0180 | [3.36, 3.43] |  | 0.972 (P-deficient) |
|  | P-deficient | 3.75 | 0.0425 | [3.66, 3.83] |  |  |
| b | Balanced | 1.00 | 0.0141 | [0.974, 1.03] | > 0.05 | 0.982 (Balanced) |
|  | P-deficient | 1.06 | 0.0237 | [1.01, 1.11] | < 0.01 |  |

**Repeating model fitting via reduced major axis regression of P weight on protein weight**

| **Parameter** | **Feed class** | **Estimate** | **95 % CI** | **Probability** | **R^2^** |
| --- | --- | --- | --- | --- | --- |
| log(a) | Balanced | -3.36 | [-3.43, -3.29] |  | 0.972 (P-deficient) |
|  | P-deficient | -3.56 | [-3.66, -3.45] |  |  |
| b | Balanced | 0.984 | [0.955, 1.01] | > 0.05 | 0.982 (Balanced) |
|  | P-deficient | 0.950 | [0.905, 0.997] | < 0.05 |  |

**Ash-Protein relationship**

**Repeating model fitting using the two variables of interest in reversed roles (e.g. with ash as the *x*-axis variable instead)**

| **Parameter** | **Feed class** | **Estimate** | **SE** | **95 % CI** | **Probability** | **R^2^** |
| --- | --- | --- | --- | --- | --- | --- |
| log(a) | Protein-deficient | 1.46 | 0.0113 | [1.43, 1.48] |  | 0.983 (Protein-deficient) |
|  | Balanced | 1.67 | 0.0104 | [1.65, 1.69] |  |  |
|  | P-deficient | 1.93 | 0.0155 | [1.90, 1.96] |  | 0.972 (Balanced) |
| b | Protein-deficient | 0.909 | 0.0230 | [0.861, 0.956] | < 0.01 |  |
|  | Balanced | 0.995 | 0.0143 | [0.967, 1.02] | > 0.05 | 0.956 (P – deficient) |
|  | P-deficient | 1.09 | 0.0299 | [1.03, 1.15] | < 0.001 |  |

**Repeating model fitting via reducing major axis regression of ash weight on Protein weight**

| **Parameter** | **Feed class** | **Estimate** | **95 % CI** | **Probability** | **R^2^** |
| --- | --- | --- | --- | --- | --- |
| log(a) | Protein-deficient | -1.58 | [-1.68, -1.49] |  | 0.983 (Protein-deficient) |
|  | Balanced | -1.65 | [-1.72, -1.58] |  |  |
|  | P-deficient | -1.76 | [-1.86, -1.65] |  | 0.975 (Balanced) |
| b | Protein-deficient | 1.09 | [1.03, 1.15] | < 0.01 |  |
|  | Balanced | 0.990 | [0.962, 1.02] | > 0.05 | 0.962 (P – deficient) |
|  | P-deficient | 0.916 | [0.870, 0.965] | < 0.01 |  |

**Supplementary Material G:** A detailed list of equations used to describe the relationships between variables in the mechanistic model

**Bodyweight composition at the start of the simulation**

The following BW partitioning was assumed to hold for a pig given a balanced, non-limiting feed prior to the start of the simulation:

- The initial empty BW was calculated as:

$$\begin{aligned} eBW\left( t_{0} \right)=\alpha\times BW\left( t_{0} \right)\#\left( G.1 \right) \end{aligned}$$

where $\alpha=0.95$ ^(1-3)^and $BW\left( t_{0} \right)$ was the initial live BW

- Consequently, $BW\left( t \right)$ was expressed as a sum of the four main components, protein ($N^{*}\left( t \right)=6.25\times N(t))$, lipid ($L\left( t \right)$), water ($W\left( t \right)$) and ash ($A\left( t \right)$)^(4)^

$$\begin{aligned} eBW\left( t \right)=N^{*}\left( t \right)+L\left( t \right)+W\left( t \right)+Ash\left( t \right)\#\left( G.2 \right) \end{aligned}$$

- The initial protein weight, ($N^{*} \left( 0 \right)$) was expressed as a percentage of $eBW\left( t_{0} \right)$:

$$\begin{aligned} N^{*}(0)=\beta\times eBW\left( t_{0} \right)\#\left( G.3 \right) \end{aligned}$$

where $\beta=0.16$ ^(5)^

- The initial weight of ash ($Ash\left( 0 \right)$), was related to $N^{*} \left( 0 \right)$ via isometry:

$$\begin{aligned} Ash\left( 0 \right)=\gamma\times N^{*} \left( 0 \right)\#\left( G.4 \right) \end{aligned}$$

where $\gamma=0.19$ ^(6)^

- The initial weight of W ($W\left( 0 \right)$), was related to $N^{*} \left( 0 \right)$ via allometry:

$$\begin{aligned} W\left( 0 \right)=\delta\times{N^{*} \left( 0 \right)}^{\epsilon} \#\left( G.5 \right) \end{aligned}$$

where $\delta=3.04\times{N_{m}^{*}}^{1-0.855}$, $\epsilon=0.855$ ^(2)^, with $N_{m}^{*}$ corresponding to the estimated mature protein weight^(7)^

- The initial L weight ($L\left( 0 \right)$), was expressed as the difference between $eBW\left( t_{0} \right)$and a sum of the remaining three main body components^(8)^:

$$\begin{aligned} L\left( 0 \right)=eBW\left( t_{0} \right)-\left( N^{*}(0)+Ash\left( 0 \right)+W\left( 0 \right) \right)\#\left( G.6 \right) \end{aligned}$$

- The initial weight of P ($P\left( 0 \right)$) was related to $N^{*}(0)$ via isometry^(9)^:

$$\begin{aligned} P\left( t_{0} \right)=\zeta\times N^{*}(0)\#\left( G.7 \right) \end{aligned}$$

where $\zeta=0.0351$, estimated on the basis of weighted linear regression of P weight on protein weight described in section 2.3

- Consequently, $P\left( t \right)$ was expressed as a sum of amounts located in soft tissue and bones^(10)^

$$\begin{aligned} P\left( t \right)=P_{soft}\left( t \right)+P_{bone}\left( t \right)\#\left( G.8 \right) \end{aligned}$$

- The initial weight of P located in bones ($P_{bone}\left( 0 \right)$), was expressed as a percentage of $P\left( 0 \right):$

$$\begin{aligned} P_{bone}\left( 0 \right)=\eta\times P\left( 0 \right)\#\left( G.9 \right) \end{aligned}$$

- The value of $\eta$ was calculated on the basis of a mean model of beta regression with a logit link function described in section 2.3:

$$\begin{aligned} \eta= \frac{\exp\left( 0.390+0.122\times\frac{P\left( 0 \right)\times1000}{eBW\left( t_{0} \right)} \right)}{\exp\left( 0.390+0.122\times\frac{P\left( 0 \right)\times1000}{eBW\left( t_{0} \right)} \right)+1} \#\left( G.10 \right) \end{aligned}$$

- The initial weight of P located in soft tissue ($P_{soft}\left( 0 \right)$), was calculated as the difference between $P\left( 0 \right)$ and $P_{bone}\left( 0 \right):$

$$\begin{aligned} P_{soft}\left( 0 \right)=P\left( 0 \right)-P_{bone}\left( 0 \right) \therefore P_{soft}\left( 0 \right)=\theta\times P\left( 0 \right)\#\left( G.11 \right) \end{aligned}$$

where $\theta=1-\eta$

**Nutrient requirements estimation**

The effective energy (EE)^(11)^ was used as the desired energy scale in the model. The EE content of feeds ($E_{feed}$; MJ/kg) was calculated as:

$$\begin{aligned} E_{feed}= 1.17\times MEC-4.29\times\left( \frac{CPC}{1000} \right)-2.4 \#\left( G.12 \right) \end{aligned}$$

where MEC is the metabolisable energy content of the feed (MJ/kg) and CPC is the crude protein content of the feed (g/kg).

- The energy maintenance requirement at time t ($E_{maint}\left( t \right)$), was estimated as a function of the current protein weight at time ($N^{*}(t)$) and mature protein weight ^(4; 12)^

$$\begin{aligned} E_{maint}\left( t \right)=1.63\times\frac{N^{*} \left( t \right)}{{N_{m}^{*}}^{0.27}} \#\left( G.13 \right) \end{aligned}$$

- The energy above $E_{maint}\left( t \right)$ needed to reach the maximum protein and L depositions at time t ($N_{max}^{*'}\left( t \right)$; $L_{max}^{'}\left( t \right)$) was calculated as^(12)^

$$\begin{aligned} E_{growth}\left( t \right)=50\times N_{max}^{*'}\left( t \right)+56\times L_{max}^{'}\left( t \right)\#\left( G.14 \right) \end{aligned}$$

- The P requirement for maintenance at time t (${P'}_{maint}\left( t \right)$; g), was estimated as a function of the current protein weight at time ($N^{*} \left( t \right)$) and mature protein weight ($N_{m}^{*}$) ^(9)^:

$$\begin{aligned} {P'}_{maint}\left( t \right)=0.1293\times\frac{N^{*} \left( t \right)}{{N_{m}^{*}}^{0.27}} \#\left( G.15 \right) \end{aligned}$$

- The maximum P retention at time t ($P_{growth_{max}}^{'}\left( t \right)$), was estimated linked to $N_{max}^{*'}\left( t \right)$ ^(9)^

$$\begin{aligned} P_{growth_{max}}^{'}\left( t \right)=\zeta\times N_{max}^{*'}\left( t \right)\#\left( G.16 \right) \end{aligned}$$

**Allocation of nutrient resources**

- The protein maintenance requirement at time t ($N_{maint}^{*'}\left( t \right)$), was estimated as a function of the current protein weight at time ($N^{*} \left( t \right)$) and mature protein weight ($N_{m}^{*}$) ^(12)^:

$$\begin{aligned} N_{maint}^{*'}\left( t \right)=0.004\times\frac{N^{*} \left( t \right)}{{N_{m}^{*}}^{0.27}} \#\left( G.17 \right) \end{aligned}$$

- The actual protein deposition at time t ($N^{*'}\left( t \right)$), was determined by the actual $FI\left( t \right)$ and calculated as^(12)^:

$$\begin{aligned} N^{*'}\left( t \right)=\left( FI(t)\times CPC\times N_{dig}^{*}\times v- N_{maint}^{*'}\left( t \right) \right)\times N_{eff}^{*} \#\left( G.18 \right) \end{aligned}$$

where $N_{dig}^{*}$ is the digestibility coefficient of crude protein, which was set to 0.8 as a default value^(13)^; *v* was the biological value of the feed^(14)^, calculated as a proportion of the most limiting amino acid (AA) in the feed relative to the ideal AA profile based on AA distribution in the body^(15)^ with a default value of 0.8^(13)^; $N_{eff}^{*}$ was the efficiency of protein utilisation for retention set to 0.763 ^(16)^

If the quantity calculated in equation (F.18) exceeded $N_{max}^{*'}\left( t \right)$, then $N^{*'}\left( t \right)= N_{max}^{*'}\left( t \right)$

- The deposition of L at time t ($L^{'}\left( t \right)$), was calculated on the basis of the energy balance, and treated as a sink^(12; 17)^

$$\begin{aligned} L^{'}\left( t \right)= \frac{FI\left( t \right)\times E_{feed}-E_{maint}\left( t \right)-50\times N^{*'}(t)}{56}\#\left( G.19 \right) \end{aligned}$$

If $L^{'}\left( t \right)<0$, then the denominator of equation (F.19) was replaced with the heat of combustion of lipid and equalled 39.6^(12)^.

- The deposition of W at time t ($W^{'}\left( t \right)$) was calculated on the basis of the relationship given in equation (F.5)^(12)^:

$$\begin{aligned} W^{'}\left( t \right)=N^{*'}\left( t \right)\times3.04\times0.855\times\left( \frac{N^{*} \left( t \right)}{N_{m}^{*}} \right)^{0.855-1} \#\left( G.20 \right) \end{aligned}$$

- The actual deposition within a body of a pig at time t $(P_{growth}^{'}\left( t \right)$), was determined by the actual $FI\left( t \right)$ and calculated as:

$$\begin{aligned} P_{growth}^{'}\left( t \right)=eff_{p}\times\left( FI\left( t \right)\times P_{feed}-P_{maint}^{'}\left( t \right) \right)\#\left( G.21 \right) \end{aligned}$$

where $eff_{p}$ is the efficiency of P utilisation for retention and set to 0.94^(9)^.

If the quantity calculated in equation (F.21) exceeded$P_{growth_{max}}^{'}\left( t \right)$, then $P_{growth}^{'}\left( t \right)= P_{growth_{max}}^{'}\left( t \right)$

- $P_{growth}^{'}\left( t \right)$ was compartmentalised and allocated to the two pools of P within the body^(10)^:

$$\begin{aligned} P_{growth}^{'}\left( t \right)={P_{growth}^{'}}_{soft}\left( t \right)+{P_{growth}^{'}}_{bone}\left( t \right)\#\left( G.22 \right) \end{aligned}$$

- The amount of P needed to support the maximum lean tissue deposition driven by $N_{max}^{*'}\left( t \right)$ at a time t was estimated as:

$$\begin{aligned} {P_{growth}^{'}}_{soft_{max}}\left( t \right)=\theta\times\zeta\times N_{max}^{*'}\left( t \right)\#\left( G.23 \right) \end{aligned}$$

- Allocation towards ${P_{growth}^{'}}_{bone}\left( t \right)$ was triggered only once both ${P'}_{maint}\left( t \right)$ and ${P_{growth}^{'}}_{soft_{max}}\left( t \right)$ were satisfied first
- The deposition of Ash at time t (${Ash}^{'}\left( t \right)$), was given by:

$$\begin{aligned} {Ash}^{'}\left( t \right)= \left\{ \begin{aligned} \kappa_{b}\times N^{*'}\left( t \right), if feed was balanced \\ \kappa_{N}\times\lambda_{N}\times N^{*'}\left( t \right)^{\lambda_{N}-1}, if feed was deficient in protein \\ \kappa_{P}\times\lambda_{P}\times N^{*'}\left( t \right)^{\lambda_{P}-1}, if feed was deficient in P \end{aligned} \right.\#\left( G.24 \right) \end{aligned}$$

where $\kappa_{b}=0.19$ ^(6)^; $\kappa_{N}=0.208$; $\kappa_{P}=0.186$; $\lambda_{N}=1.08$; $\lambda_{P}= 0.873$, estimated on the basis of statistical analyses outlined in section 2.3.

**References**

1. Moughan PJ, Smith WC, Pearson G (1987) Description and validation of a model simulating growth in the pig (20–90 kg liveweight). *New Zeal J Agr Res* **30**, 481-489.

2. Emmans GC, Kyriazakis I (1995) A general method for predicting the weight of water in the empty bodies of pigs. *Animal Sci* **61**, 103-108.

3. Whittemore EC, Emmans GC, Kyriazakis I (2003) The problem of predicting food intake during the period of adaptation to a new food: a model. *Br J Nutr* **89**, 383-399.

4. Emmans GC, Fisher C (1986) Problems in nutritional theory. In *Nutrient requirements of poultry and nutritional research*, vol. 19, pp. 9-39 [GC Emmans and C Fisher, editors]. London, UK: Butterworths & Co (Publishers) Ltd, .

5. Kyriazakis I, Whittemore CT (2006) *Whittemore's Science and Practice of Pig Production*. 3 ed*.* Oxford, UK: Blackwell Publishing

6. Emmans GC, Kyriazakis I (1997) Models of pig growth: problems and proposed solutions. *Livest Prod Sci* **51**, 119-129.

7. Wellock IJ, Emmans GC, Kyriazakis I (2004) Describing and predicting potential growth in the pig. *Animal Sci* **78**, 379-388.

8. Filipe JAN, Leinonen I, Kyriazakis I (2018) The quantitative principles of animal growth. In *Feed Evaluation Science*, 1 ed. [PJ Moughan and WH Hendriks, editors]. Wageningen: Wageningen Academic Publishers.

9. Symeou V, Leinonen I, Kyriazakis I (2014) Modelling phosphorus intake, digestion, retention and excretion in growing and finishing pigs: model description. *Animal* **8**, 1612-1621.

10. Crenshaw TD (2001) Swine Nutrition. In *Calcium, Phosphorous, Vitamin D, and Vitamin K in Swine Nutrition*, 2 ed., pp. 187 - 209 [SLL Lewis A.J., editor]. N.W. Corporate Blvd., Boca Raton, Florida, USA: CRC Press.

11. Emmans GC (1994) Effective energy: a concept of energy utilization applied across species. *Br J Nutr* **71**, 801-821.

12. Wellock IJ, Emmans GC, Kyriazakis I (2003) Modelling the effects of thermal environment and dietary composition on pig performance: model logic and concepts. *Animal Sci* **77**, 255-266.

13. Wellock IJ, Emmans GC, Kyriazakis I (2003) Modelling the effects of thermal environment and dietary composition on pig performance: model testing and evaluation. *Animal Sci* **77**, 267-276.

14. Wang TC, Fuller MF (1989) The optimum dietary amino acid pattern for growing pigs: 1. Experiments by amino acid deletion. *Br J Nutr* **62**, 77-89.

15. National Reserach Council (2012) *Nutrient Requirements of Swine*. 11 ed*.* Washington, D.C., USA: The National Academies Press.

16. Sandberg FB, Emmans GC, Kyriazakis I (2005) Partitioning of limiting protein and energy in the growing pig: description of the problem, possible rules and their qualitative evaluation. *Br J Nutr* **93**, 205-212.

17. Ferguson NS, Gous RM, Emmans GC (1994) Preferred components for the construction of a new simulation model of growth, feed intake and nutrient requirements of growing pigs. *S Afr J Anim Sci* **24**, 10-17.

**Supplementary Material H:** Scaled residuals (expressed as the difference between the reported and predicted data scaled by the observed standard deviations) against simulated predictions from the mechanistic model of P utilisation for: 1) daily Phosphorus (P) deposition (g/day); 2) daily protein deposition (g/day); 3) average daily feed intake (ADFI; kg/day). Residuals corresponded to simulations from the three papers: 1) Adeola *et al*.; 2) Pomar *et al*.; and 3) Ekpe *et al*.

| **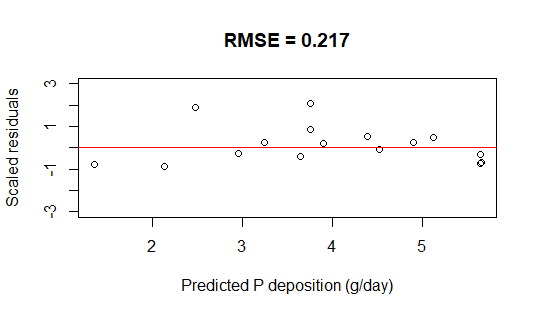** | **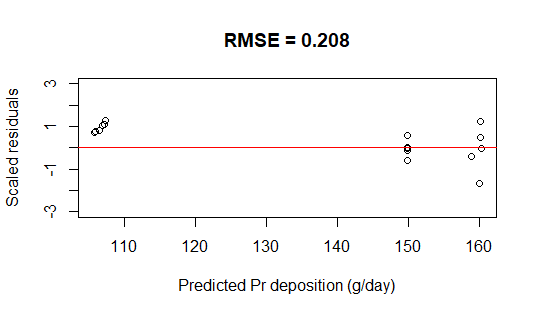** |
| --- | --- |
| **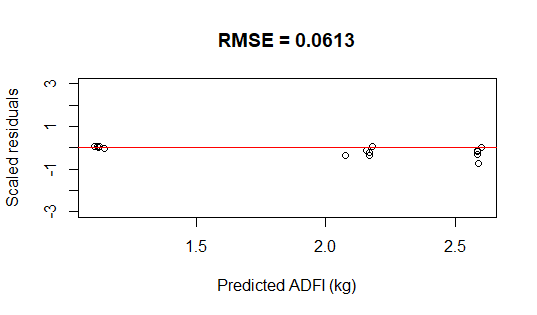** |  |
